# AI-driven tumor heterogeneity quantification and survival prediction in pancreatic ductal adenocarcinoma

**DOI:** 10.1038/s41746-026-02979-7

**Published:** 2026-07-20

**Authors:** Lizhi Shao, Xinyi Ke, Yun Wang, Ruiyu Li, Kedian Yu, Junxian Wu, Jingci Chen, Ruping Hong, Zheng Wang, Junliang Lu, Hui Zhang, Jianru Sun, Ziyu Zhou, Xiaoming Jiang, Lianghua He, Hongyu Zhou, Tao Chen, Yueping Liu, Jie Tian, Huanwen Wu, Zhiyong Liang

**Affiliations:** 1 School of Computer Science and Technology, Tongji University, Shanghai, China; 2 Department of Pathology, State Key Laboratory of Common Mechanism Research for Major Diseases, Peking Union Medical College Hospital, Chinese Academy of Medical Sciences and Peking Union Medical College, Beijing, China; 3 Department of Pathology, the First Medical Center of People’s Liberation Army General Hospital, Beijing, China; 4 The First Clinical Medical College of Anhui Medical University, Hefei, China; 5 Chongqing Engineering Research Center of Medical Electronics and Information Technology, Chongqing, University of Post and Telecommunications, Chongqing, China; 6 Department of Automation, Tsinghua University, Beijing, China; 7 Department of Gastrointestinal Hernia Surgery, Southern Hospital Ganzhou Hospital, Ganzhou, China; 8 Department of Pathology, The Fourth Hospital of Hebei Medical University, Shijiazhuang, China; 9 CAS Key Laboratory of Molecular Imaging, Institute of Automation, Beijing, China; 10 Beijing Advanced Innovation Center for Big Data-Based Precision Medicine, School of Engineering Medicine, Beihang University, Beijing, China and Key Laboratory of Big Data-Based Precision Medicine (Beihang University), Ministry of Industry and Information Technology of the People’s Republic of China, Beijing, China

**Keywords:** Biomarkers, Cancer, Computational biology and bioinformatics, Oncology

## Abstract

Pancreatic ductal adenocarcinoma (PDAC) is one of the most lethal malignancies worldwide, and accurate prognostic prediction remains highly challenging due to its marked biological heterogeneity and complex tumor microenvironment. To address this challenge, a histopathomics-based survival prediction system (HPSurv) was developed using histopathological whole-slide images (WSIs) for individualized overall survival (OS) prediction. Within this framework, pathological tissue classification, quantitative characterization of tumor spatial heterogeneity, and a survival Transformer were integrated to enable multi-level representation learning from histopathological data. The system was developed and evaluated in 1020 patients across five independent cohorts. Compared with conventional clinicopathological indicators, significantly improved prognostic performance was achieved across multicenter cohorts (*p* < 0.05), with a mean C-index of 0.761 and time-dependent AUCs of 0.936, 0.877, and 0.772 for predicting 6-month, 2-year, and 3-year survival, respectively. Subgroup analyses further supported its role as an independent prognostic factor and suggested its potential utility in stratifying patients with respect to ACT-related outcomes. In addition, significant associations with key PDAC molecular pathways were observed, providing biological insights into the model predictions and supporting interpretability. In the study, an interpretable and high-performing artificial intelligence (AI) framework for quantitative modeling of PDAC was established. Objective characterization of tumor heterogeneity and accurate postoperative survival prediction are enabled, with potential value for personalized management in PDAC.

## Introduction

Pancreatic ductal adenocarcinoma (PDAC) is among the most lethal solid tumors, with only a 13% 5-year overall survival rate^1^. Radical surgical resection remains the sole option for treatment with proven curative potential^2–4^. Nevertheless, nearly 70% of patients succumb to the disease within three years after surgery^5^. Although adjuvant chemotherapy (ACT) significantly prolongs survival and is recommended for all resected cases by current National Comprehensive Cancer Network (NCCN) guidelines^6^, the treatment is frequently curtailed by toxicities^7^, and a sizeable subset of patients derives little benefit because of their intrinsic chemoresistance^8^. Consequently, robust postoperative risk stratification is essential for enabling truly personalized therapy.

Overall survival (OS) or disease-free survival (DFS) is commonly used in clinical practice as endpoints to evaluate the efficacy of postoperative adjuvant treatment in PDAC patients. There are various postoperative adjuvant chemotherapy (ACT) for PDAC, wherein single or combination chemotherapy primarily based on fluorouracil or gemcitabine is the most widely used^9,10^. Additionally, the mFOLFIRINOX regimen (a four-drug combination) offers greater benefits compared to classic regimens. Under this regimen, the median DFS of patients is extended by 70%, whereas the 2-year disease-free survival rate increases by 16%^11,12^. However, the increased chemotherapy toxicity of mFOLFIRINOX has prompted clinicians to exercise great caution in its use, particularly for patients with poor physical condition^13^. From the perspectives of disease progression, complications, and patient care, postoperative clinical strategies that involve a “wait-and-see” strategy are also a viable option^14^. Furthermore, reliable prognostic prediction tools hold promise for providing additional supportive information when developing postoperative ACT plans^15–17^.

The tumor spatial heterogeneity of PDAC reflects molecular characterization differences among patients that impact their overall outcomes^18^. Extensive and significant tumor stroma is one of the most distinctive pathological features of pancreatic cancer^19,20^. Consequently, histopathology remains the cornerstone of PDAC risk assessment for analysis^21,22^. Researchers have found that factors such as the area, proportion, and classification of different pathological tissue types within the tumor are significantly associated with PDAC prognosis and treatment efficacy^23,24^. Although whole slide imaging (WSI) provides a broad-field, high-magnification data source for tumor tissue evaluation, performing pixel-level annotation and quantifying histopathological information across the entire tumor slide is an extremely challenging task, with limited clinical applicability^25,26^. Additionally, integrating quantified histopathological information into the modeling process may offer more precise patient risk stratification tools based on a more complete description of tumor heterogeneity^27,28^.

The emergence of AI has yielded a feasible solution to this challenge. Deep learning models have improved the model’s ability to differentiate between tissues with significant visual differences^27,29,30^. The recently established pathology foundation models were more powerful in this regard^31–34^. Similarly, pixel-level image feature encoders provide clearer sparse representations of histopathological information, which further enhance model performance in tasks such as tissue classification, diagnosis, and grading^35^. Accurate tissue classification provides a strong foundation for the quantification of histopathology. For prognosis prediction, multi-instance learning networks, transformers, and graph convolutional neural network have all been proven effective^30^. ML-based pathomics methods are among the AI methods that balance pathology interpretability^28^. Higher accuracy of performance, more stable cross-center performance, and stronger interpretability are the key requirements for efficient AI-based predictive models that can accelerate clinical translation^36^.

**Fig. 1:**
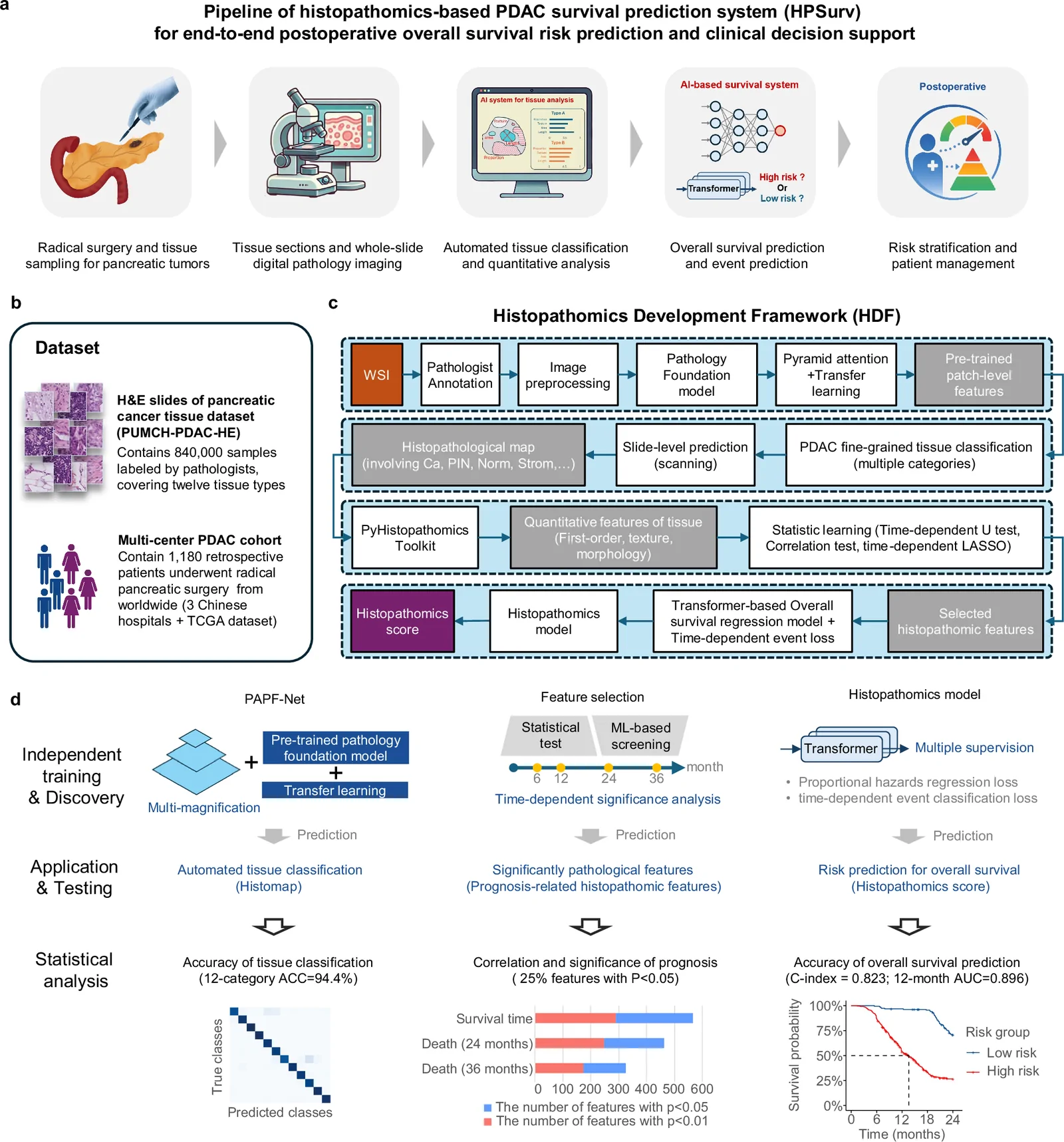
Illustration of proposed end-to-end prognosis prediction for pancreatic ductal adenocarcinoma (PDAC) overall survival (OS). **a** Pipeline of histopathomics-based PDAC survival prediction system (HPSurv). **b** Image datasets and multicenter clinical cohorts. **c** Histopathomics development framework (HDF) for HPSurv development, including tissue classification, histopathomic feature extraction, and histopathomics model building. **d** Development and testing of HPSurv for pathological tissue classification, OS-related significant features screening, and OS prediction.

This study proposed a histopathomics-based PDAC survival prediction system (HPSurv) using histopathological images for postoperative OS prediction (Fig. 1a). For development and validation, a pancreatic tumor histopathology image dataset and multicenter cohort of PDAC were constructed, comprising 840,000 samples labeled by pathologists and real-world data from 1020 patients (Fig. 1b). This system aimed to develop a reliable predictive tool using histopathological WSI for OS risk in PDAC in more refined patient management, and was implemented through the integration of pathological tissue classification (PAPF-Net), quantification of histopathological heterogeneity (histopathomic features), and survival prediction (histopathomics model) (Fig. 1c, d). The performance of HPSurv was evaluated across multiple centers to enhance confidence in clinical implementation.

## Results

### Patient characteristics

A total of 1020 patients with PDAC and their corresponding data were collected for model development, independent internal validation, and external testing (Supplementary Fig. 1). These patients were obtained from five hospitals in China and one international public dataset, and were designated as the primary cohort (PC), validation cohort (VC), and external testing cohorts (ExTC1-4), respectively. The clinical characteristics of the enrolled patients are summarized in Supplementary Table 1. No significant differences were observed in sex distribution or pathological T stage across the multicenter cohorts (*P* > 0.05). The median follow-up time for the primary cohort was 47 months, with a median overall survival of 31 months. The median follow-up and survival times for the multicenter cohorts are provided in Supplementary Table 2. Furthermore, the American Joint Committee on Cancer (AJCC) 8th edition staging system was applied and reassessed to enable more comprehensive subgroup and stratified analyses. In this study, the primary cohort was used for feature selection and model training, while the remaining cohorts were used exclusively for independent validation or testing.

### Pathological tissue classification of pancreatic ductal adenocarcinoma in a whole slide image

**Fig. 2:**
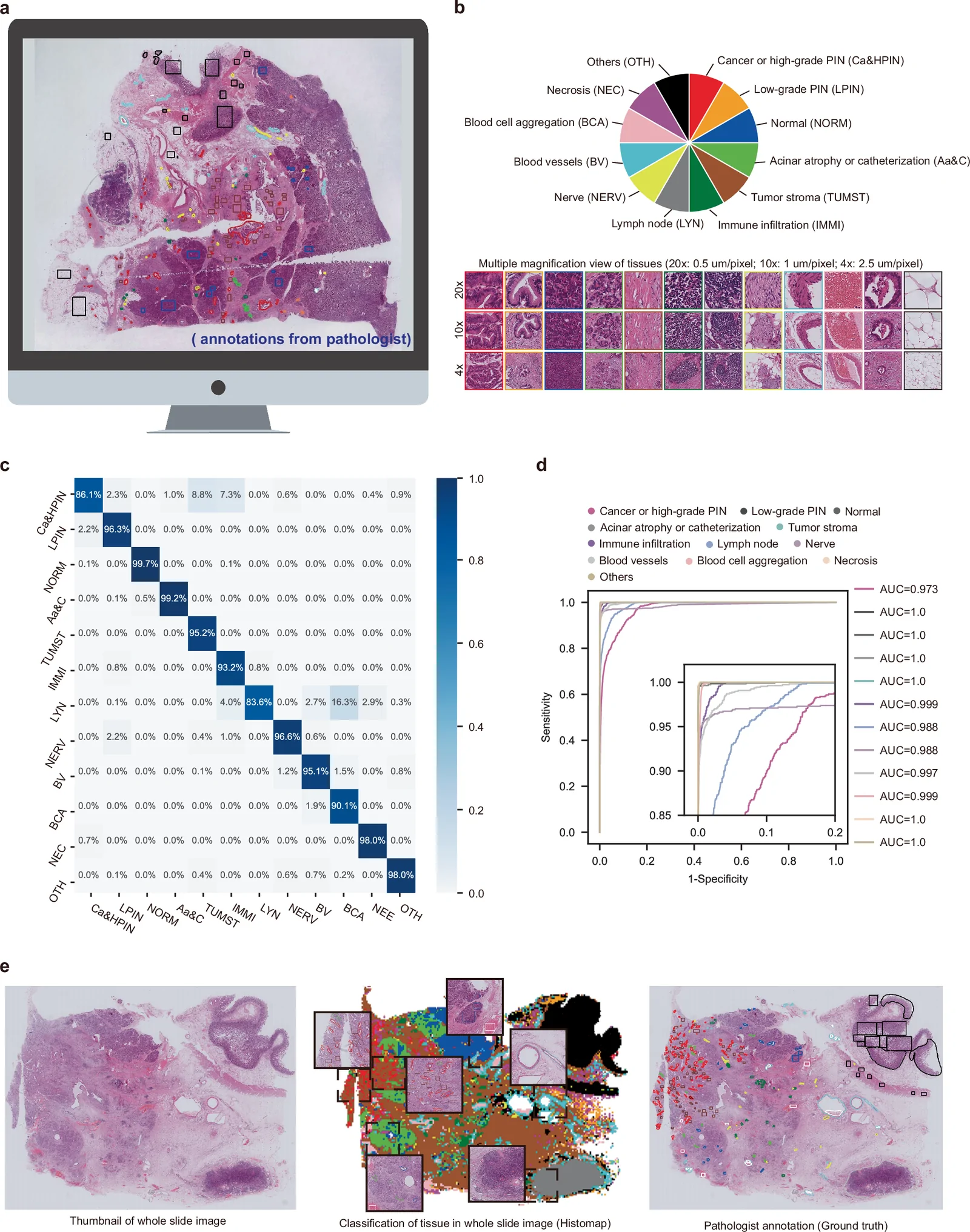
PDAC tissue classification. **a** Expert-level annotations of representative regions in PDAC pathological tissue. **b** Twelve annotation categories with multiple magnification examples of PDAC pathological tissue. **c** Confusion matrix for PDAC tissue classification using the pyramid attention-based pathology foundation model transfer learning network (PAPF-Net). **d** Receiver operating characteristic curve (ROC) analysis evaluating the ability of PAPF-Net to discriminate between target tissue categories and the control group. **e** Fully automated tissue classification and construction of PDAC histopathological map (Histomap) for whole-slide images.

Pathological tissue classification was the basis for describing the spatial heterogeneity of tumors through histopathological images. A pyramid attention-based pathology foundation model transfer learning network (PAPF-Net) was constructed via transfer learning based on a pathological foundation model, in conjunction with pyramid attention fusion to overcome the challenges of pancreatic tumor tissue classification—including cancer or high-grade Pancreatic Intraepithelial Neoplasia (PIN), low-grade PIN, normal, acinar atrophy or catheterization, tumor stroma, immune infiltration, lymph node, nerve, blood vessels, blood cell aggregation, necrosis, and others (Fig. 2 and Supplementary Table 3).

Twelve types of pathological tissues were distinguished by PAPF-Net with a multi-classification accuracy (ACC) of 0.944 [0.942–0.946]. Additionally, the average precision and recall of PAPF-Net were 0.948 [0.946–0.95] and 0.944 [0.942–0.946], respectively, whereas the F1-score for the performance of PAPF-Net was 0.943 [0.941–0.944]. Regarding the performance in distinguishing target tissues from control groups, PAPF-Net achieved an AUC greater than 0.97 and ensured reliable sensitivity as well as specificity (Supplementary Table 4).

Pyramid input and information fusion capture various features such as cellular traits, cell clustering, tissue architecture, and morphology, which make the approach adaptable to different tissue identification scenarios. For instance, cancer and high-grade PIN rely largely on high-resolution views. In contrast, tumor stroma requires not only high-resolution details, but also observations of tissue distribution on a larger scale. Additionally, transfer learning based on the pathological foundation model enhanced performance of classification (Supplementary Table 5).

### Histopathological heterogeneity quantification of pancreatic ductal adenocarcinoma

**Fig. 3:**
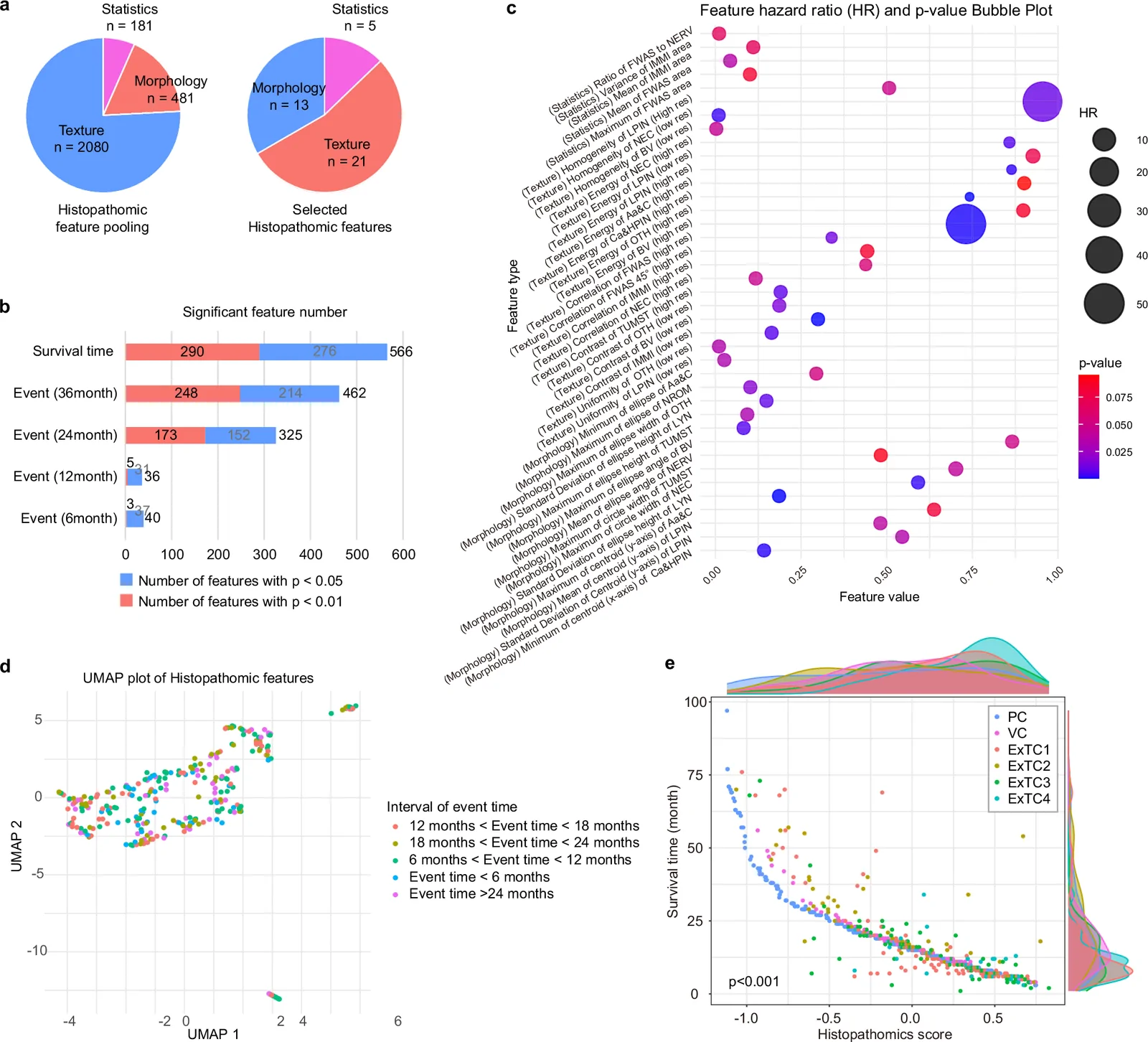
Correlation analysis between histopathomic features for progosis. **a** Distributions of histopathomic features. **b** The statistical significance of the correlation between histopathomic features and overall survival (OS). **c** Bubble plot for selected histopathomic features of hazard ratio and significance in the OS regression analysis. **d** Uniform manifold approximation and projection (UMAP) analysis for histopathomic features under different time-dependent event (death). **e** Distributional consistency test between the signatures generated by selected histopathomic features and overall survival.

A total of 2742 histopathomic features were extracted from the WSI to quantify tissue characteristics (Fig. 3a), which collectively described the spatial heterogeneity of PDAC. Of these, 75.9% were related to tissue texture, which is closely associated with contrast, uniformity, complexity, and smoothness of tissue region pixels. Additionally, 17.5% represented morphological information, including perimeter, convexity, radius, and centroid, whereas 6.6% were related to statistical features describing tissue area, absolute proportion, relative ratio, and tissue category diversity. Univariate analysis revealed that 566 (20.6%) features were significantly associated with overall survival in PDAC (*p* < 0.05), with 290 (10.6%) features exhibiting significance values below 0.01 (Fig. 3b). The extracted histopathomic features were significantly associated with time-dependent events (death) in PDAC patients across 6-month, 12-month, 24-month, and 36-month intervals.

Following OS-related screening, 39 features were selected that represented various pathological tissue types, including cancer, tumor stroma, nerves, immune infiltration, and low-grade PIN. Of these, 53.8% were related to tissue texture, 33.3% to morphological characteristics, and 12.8% to statistical features, such as nerve proportion, immune infiltration area, and total tissue area. These selected histopathomic features were significantly associated with OS survival (Fig. 3c). However, they were still insufficient for accurately differentiating time-dependent events in patients, as demonstrated by the clustering results (Fig. 3d). The histopathomics model demonstrated strong consistency with actual OS survival across multiple centers (*p* < 0.001) and a negative correlation with survival months (Fig. 3e).

### Performance of the histopathomics model in overall survival prediction in pancreatic ductal adenocarcinoma

**Fig. 4:**
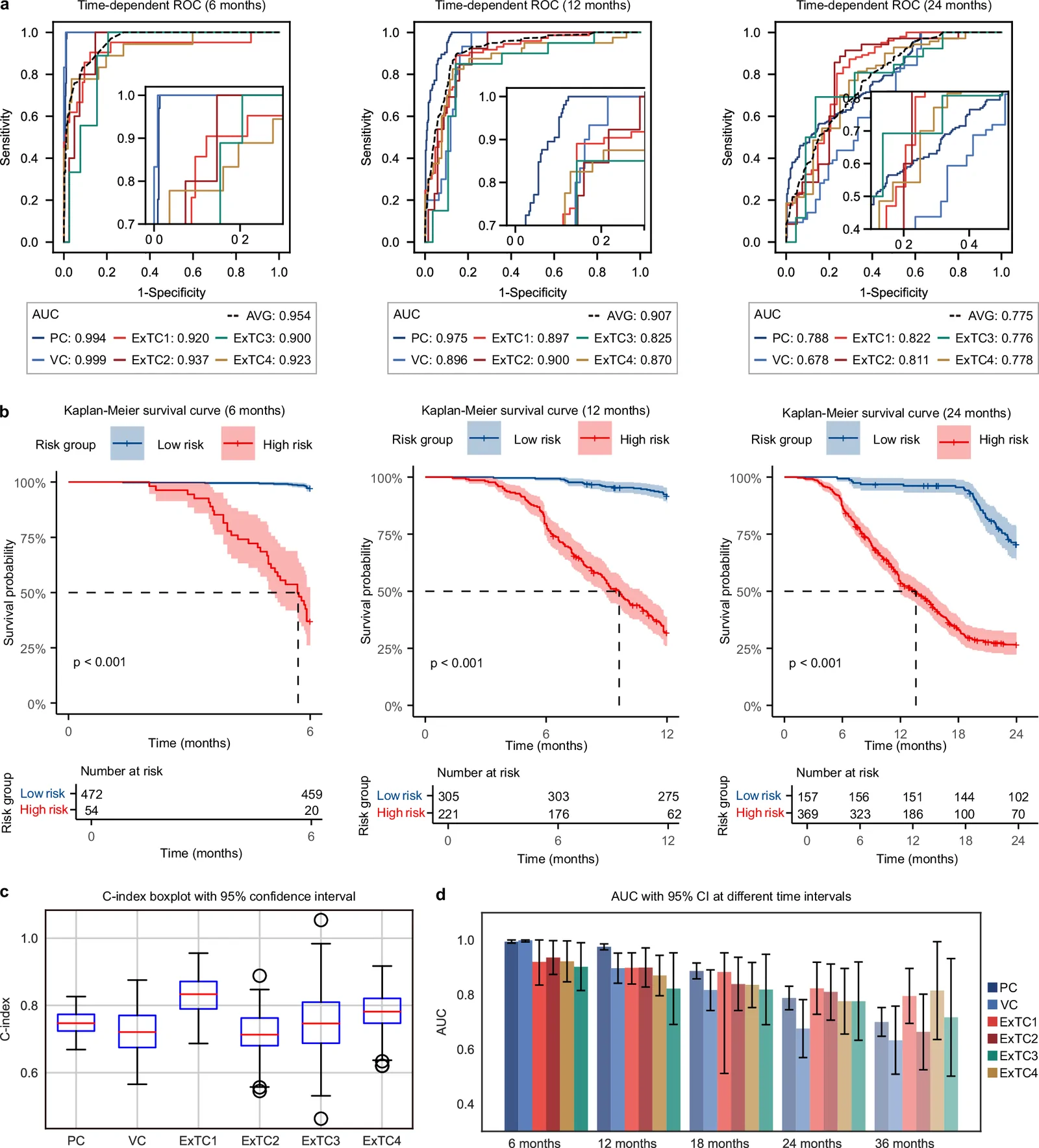
Performance of histopathomics model for prognosis prediction among multiple centers. **a** Receiver operating characteristic curve (ROC) of the histopathomics model for time-dependent event (death) prediction. **b** Kaplan-Meier (KM) survival curve of the histopathomics model for overall survival (OS) regression prediction. **c** Robustness analysis of the histopathomics model for OS regression prediction among multiple centers using Concordance index. **d** Robustness analysis of the histopathomics model for time-dependent event (death) prediction among multiple centers using area under the curve (AUC). The significance between high and low risk group was evaluated by log-rank test.

The overall performance of the histopathomics model revealed significant correlation with the survival term and time-dependent death (Supplementary Table 6). The predicted values demonstrated significant distributional consistency with actual OS rates. Furthermore, the histopathomics model yielded a C-index of 0.750 [0.718–0.783] in the PC and 0.726 [0.658–0.795] in the VC. During multi-center testing, the performance of the histopathomics model maintained a C-index of 0.823 [0.769–0.878], 0.712 [0.632–0.792], 0.782 [0.719–0.846], and 0.748 [0.654–0.841] in ExTC1, ExTC2, ExTC3, and ExTC4, respectively (Fig. 4a, b, c). Moreover, time-dependent analysis of histopathomics model was implemented for time intervals of 6-, 12-, 24-, and 36-month, to account for predictions of short-, short medium-, medium-, and long-term survival probabilities. The average AUC for short-term (< 6 months) death prediction across multiple centers was 0.920 [0.843–0.997], with high negative predictive value (NPV), thereby underscoring the reliability of the model for treatment decision-making in short-term death predictions. The prediction accuracy of the model demonstrated a slight decline with an increase in the prediction time endpoint; however, it still maintained an AUC greater than 0.8 at 18 months. The variations in sensitivity, specificity, positive predictive value (PPV), and NPV with respect to the prediction time endpoints are presented in Supplementary Table 7. The histopathomics model consistently exhibited high PPV and sensitivity across all time-dependent event predictions. Stain variability and batch effects of the model were also analyzed (Supplementary Table 8). Only diagnostic slides were analyzed for each patient, while different scanner platforms were used across participating centers. The consistent external validation results support the robustness of the model to scanner heterogeneity and its generalizability across independent cohorts. Benchmarking against baseline approaches for OS prediction was listed in Supplementary Table 9.

Moreover, the time-dependent receiver operating characteristic curve (ROC) demonstrated the ability to predict death events within fixed time intervals, thus providing greater confidence in using predictions for adjunctive treatment planning (Fig. 4a). The Kaplan–Meier (KM) curve analysis revealed the capacity to distinguish between high and low death risk groups among patients with postoperative PDAC (Fig. 4b). Additionally, the decision curve demonstrated the clinical benefit predicted by HPSurv compared with no intervention (Supplementary Fig. 2). The calibration curve indicated the alignment of the predicted probabilities with the true event probabilities (Supplementary Fig. 3). Overall, the model maintained its reliability and robustness in multi-center testing (Fig. 4c and Supplementary Fig. 4). For long-term survival prediction, the reliability slightly fluctuated as survival duration increased in the multi-center test (Fig. 4d).

### Comparison between clinical indicators and HPSurv for overall survival prediction

**Fig. 5:**
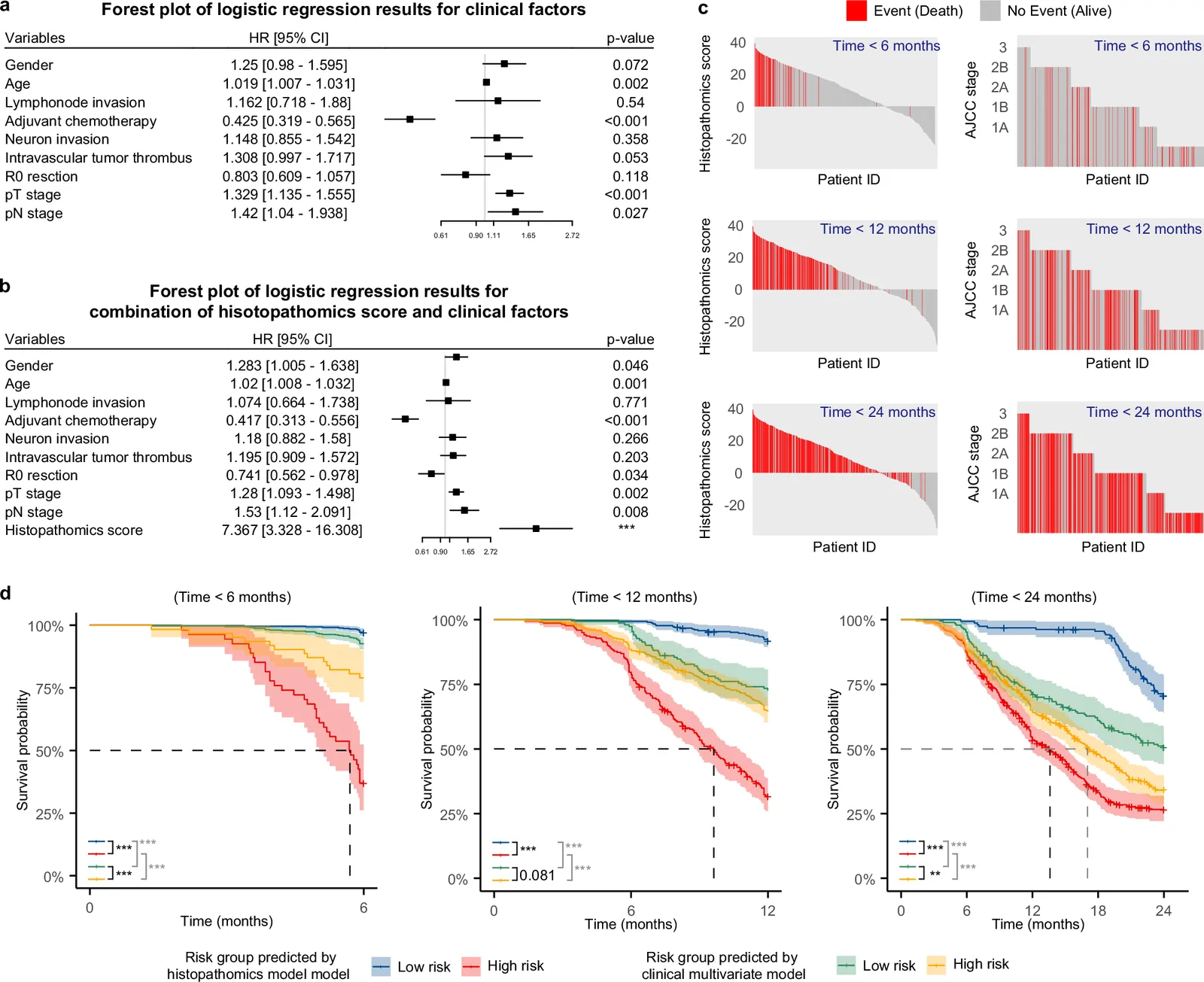
Analysis of clinical factors for overall survival (OS) association and comparison. **a** Forest plot based on multivariate analysis of clinical factors for OS regression. **b** Forest plot based on multivariate analysis of integrating clinical factors and HPSurv for OS regression. **c** Waterfall plot of American Joint Committee on Cancer (AJCC) stage and HPSurv in time-dependent OS predictions. **d** Kaplan–Meier survival curve analysis for comparison between clinical multifactorial model and HPSurv at different time intervals. The clinical multivariable model was developed using a Cox proportional hazards (CPH) model based on gender, age, lymph node status, neural invasion, intravascular tumor thrombus, negative resection margin, pathological T stage, and pathological N stage. In analyses involving clinical variables, median imputation was applied for missing data in joint modeling with HPSurv, while only patients with complete clinical data were included in comparative analyses to minimize selection bias. The significance tested by log-rank test: *, *p* < 0.05; **, *p* < 0.001; ***, *p* < 0.0001.

**Fig. 6:**
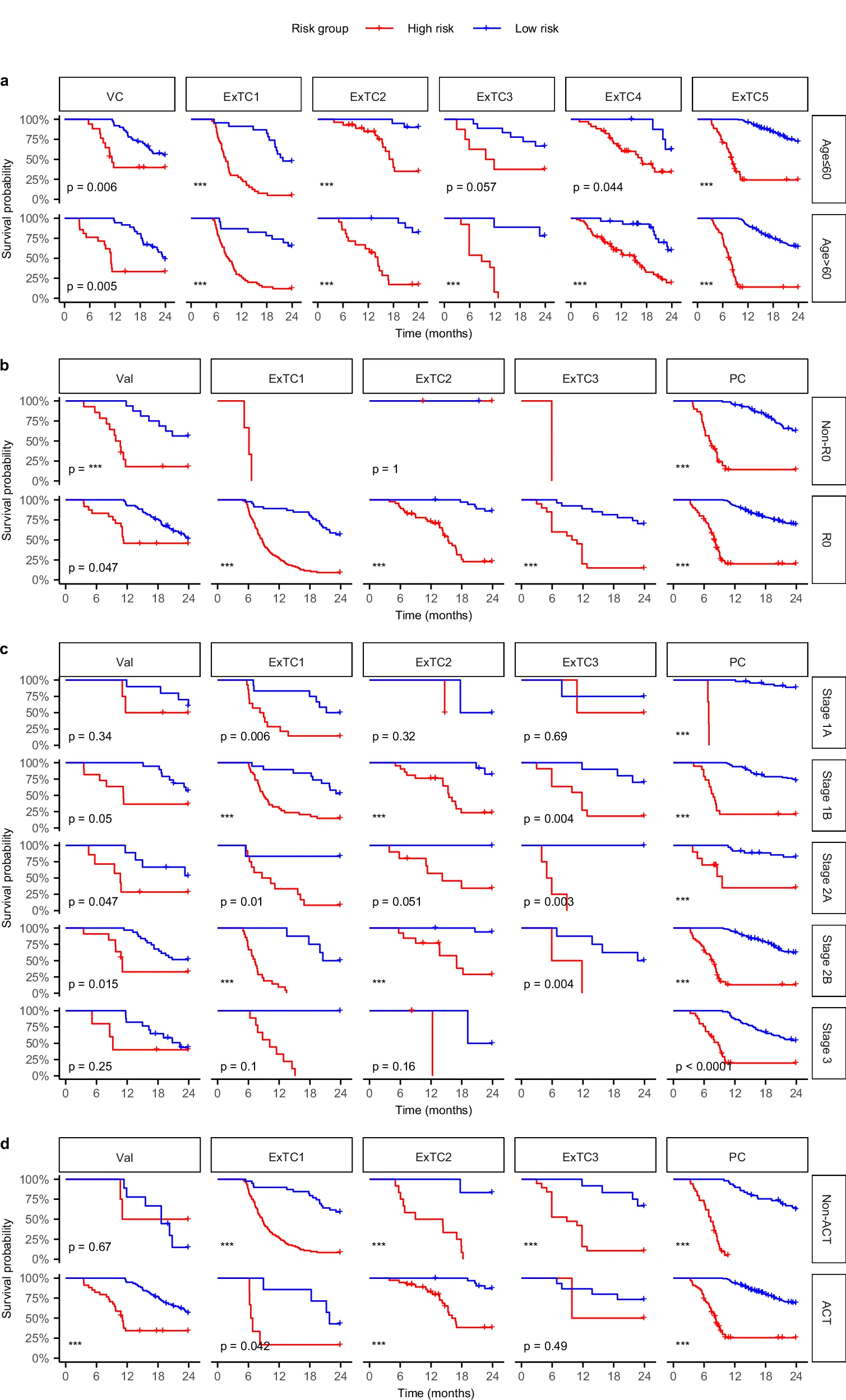
Clinical subgroup analysis of HPSurv for overall survival (OS) prediction by Kaplan–Meier survival curve. **a** Age. **b** R0 resection. **c** Stage of the eighth edition American Joint Committee on Cancer (AJCC). **d** Postoperative adjuvant chemotherapy. ***, *p* < 0.0001.

Clinical variables considered to be associated with prognosis in PDAC included lymph node invasion, perineural invasion, intravascular tumor thrombus, R0 resection, pathological T stage, pathological N stage, and AJCC (8th edition) stage. As the basis for prognostic risk assessment in clinical practice, clinical indicators for PDAC were subjected to univariate analysis for OS prediction across multiple centers. Individual clinical variables showed limited and unstable performance for OS prediction across multicenter cohorts, with mean C-index values below 0.6. (Supplementary Tables 10–16). Multivariate analysis was performed to clarify the relative independence of these clinical indicators and the effects of their interaction with OS rates (Fig. 5a). Both univariate and multivariate analyses were based on the Cox proportional-hazards (CPH) model. Postoperative adjuvant chemotherapy and negative resection margin were identified as significant protective factors, whereas all the remaining factors were associated with increased risk. The histopathomics score of HPSurv was included in the multivariate analysis and exhibited the highest hazard ratio (HR = 7.367 [3.328–16.308]). Compared to the clinical variables, the HPSurv demonstrated the strongest independent prognostic value (Fig. 5b). According to the waterfall plot, the histopathomics score revealed greater consistency between the risk score and survival outcomes at different time points compared to the AJCC staging (Fig. 5c). Furthermore, the HPSurv exhibited a superior risk stratification ability compared to the clinical multivariable model (Fig. 5d). The model combining histopathomics score and clinical information demonstrated an improvement over the clinical multivariate survival regression model (Supplementary Table 17). However, the combination model failed to further enhance the predictive performance of the histopathological model. Additionally, the performance of HPSurv was evaluated in clinical subgroups for adaptive subpopulation selection (Fig. 6 and Supplementary Fig. 5), wherein it remained robust across multiple centers, areas and populations.

### Exploratory analysis of postoperative adjuvant chemotherapy-associated outcome patterns across HPSurv-defined risk groups

An exploratory analysis was conducted to examine the association between HPSurv-defined risk groups and clinical outcomes among patients receiving different postoperative ACT regimens (Supplementary Fig. 6a,b). Patients classified as high-risk by HPSurv exhibited larger outcome differences between ACT-treated and non-ACT-treated groups than those classified as low-risk. In contrast, outcome differences associated with ACT status were less apparent in the low-risk group. AJCC stage is widely used for clinical risk stratification; however, limited separation of ACT-associated outcome patterns was observed across AJCC-defined subgroups, except for stage IA (Supplementary Fig. 6c).

**Fig. 7:**
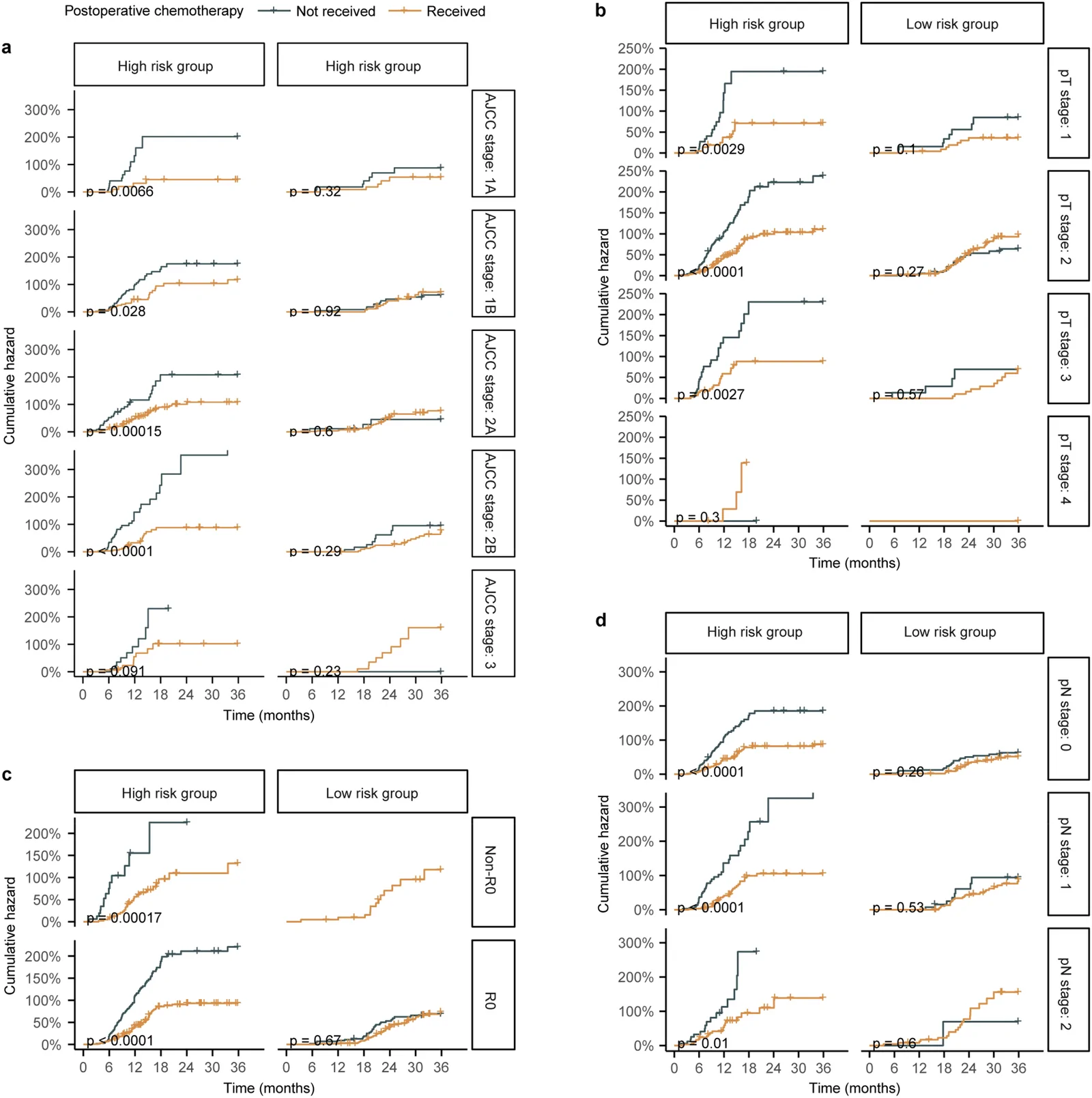
Stratified analysis based on HPSurv for postoperative adjuvant chemotherapy in the prognosis-related clinical group. **a** American Joint Committee on Cancer (AJCC) stage. **b** Pathological T stage. **c** Negative surgical margins (R0 resection). **d** Pathological N stage.

Further stratified analyses combining HPSurv predictions with clinicopathological variables were performed to characterize outcome patterns across patient subgroups (Fig. 7). Within the HPSurv-defined low-risk group, similar outcome patterns were observed regardless of ACT status across AJCC stage, pathological T stage (pT), and pathological N stage (pN). In contrast, greater outcome differences between ACT-treated and non-ACT-treated patients were observed in the HPSurv-defined high-risk group. These findings suggest that HPSurv captures prognostically relevant heterogeneity among patients; however, the retrospective design of the study precludes conclusions regarding the predictive value of HPSurv for chemotherapy benefit.

### Interpretability analysis of HPSurv

The interpretability of the model was illustrated using the histopathological tissue map (Histomap; Fig. 2e), which highlights regions used for feature extraction (Supplementary Fig. 7). Representative computational visualizations are presented in Supplementary Figs. 8 and 9. The semantic meaning of these features is detailed in Supplementary Table 16, and prognostic relevance and importance are visualized using a bubble plot (Fig. 3c). Feature importance was also evaluated by SHapley Additive exPlanations (SHAP) Analysis (Supplementary Fig. 10).

To demonstrate that the HPSurv system captured meaningful tumor information rather than isolated data fitting, multi-omics data were integrated to validate the deep learning model. It was investigated whether the histopathomic features from HPSurv underlying the risk scores could serve as morphological surrogates for known oncogenic pathways and driver mutations. Firstly, transcriptomic analysis was performed using the TCGA-PAAD cohort (*n* = 127). A total of 350 upregulated and 93 downregulated genes were identified in the high-mortality risk group compared with the low-mortality group (Supplementary Fig. 11a). To ensure the robustness of these findings, multiple testing correction was applied (Benjamini–Hochberg [BH] adjusted *P* < 0.05). Functional enrichment analysis revealed that high-risk predictions were significantly associated with dysregulation in metabolic and immune-related processes. Specifically, enriched Kyoto Encyclopedia of Genes and Genomes (KEGG) pathways and Gene Ontology (GO) terms included pancreatic secretion, keratinocyte differentiation, immune response, and lipid metabolism (Supplementary Fig. 11b, c). Furthermore, protein–protein interaction (PPI) network analysis identified tightly connected gene clusters associated with keratinization (KRT5, KRT14) and immune response regulation (CD19, CD79A), particularly B-cell-related pathways (Supplementary Fig. 11d). These findings suggest that the HPSurv captures key transcriptomic characteristics of an aggressive tumor microenvironment.

**Fig. 8:**
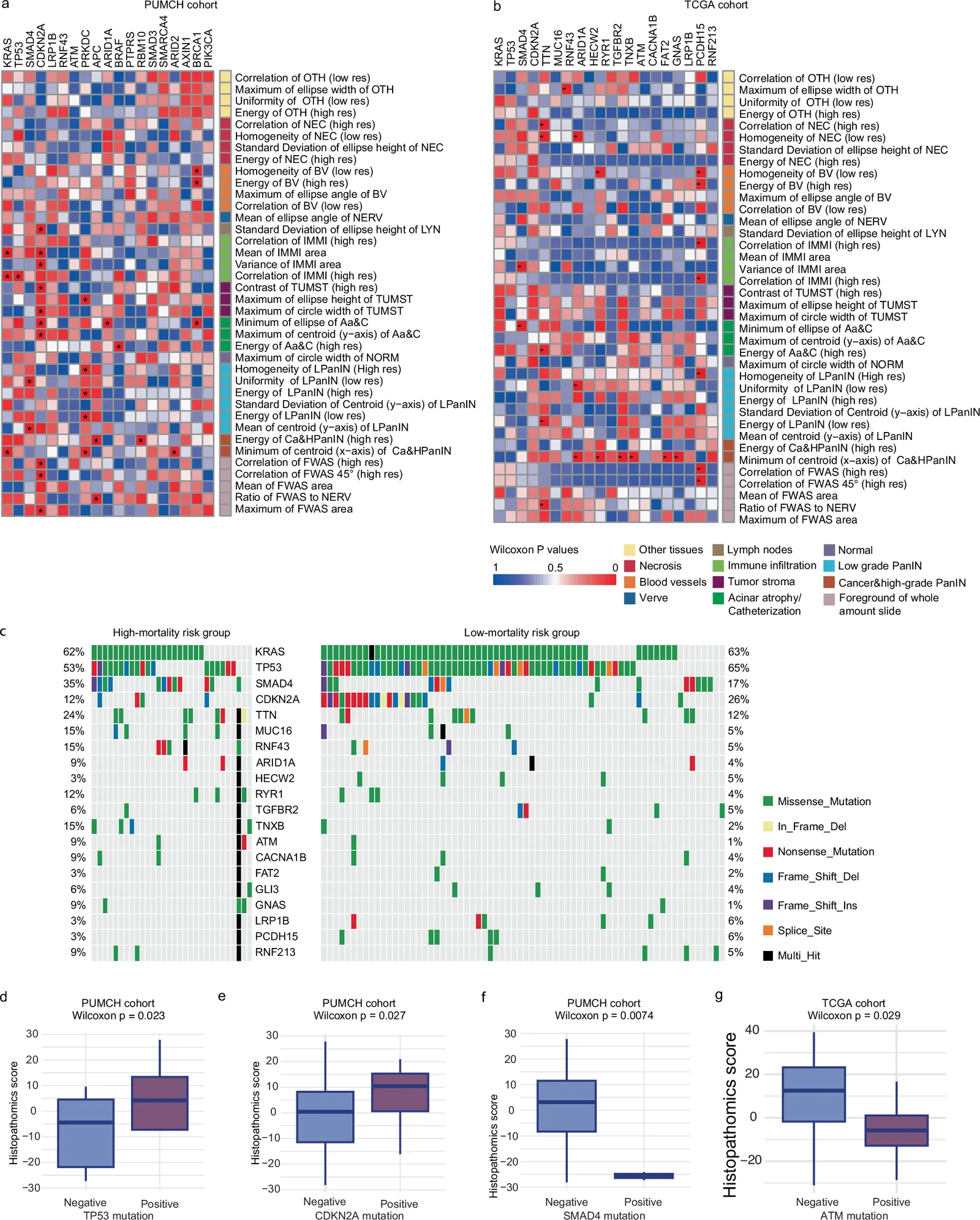
Associations between HPSurv and gene mutations. Wilcoxon rank-sum tests are used to evaluate whether histopathomic features of HPSurv differ between patients with or without mutations of key driver genes in the PUMCH cohort (**a**) and in the TCGA cohort (**b**). Asterisk means *p* values less than 0.05. **c** The oncoplot displays the mutational landscape of high-mortality and low-mortality risk groups. **d**-**g** Bar plots show that there exist different levels of HPSurv prediction between patients with or without TP53 (**d**), CDKN2A (**e**), SMAD4 (**f**) mutations in the PUMCH cohort, and ATM (**g**) Mutation in the TCGA cohort, using Wilcoxon rank-sum tests.

Next, genomic alterations associated with HPSurv were evaluated in both the PUMCH (*n* = 82) and TCGA-PAAD (*n* = 127) cohorts. Wilcoxon rank-sum tests were employed to assess the associations between genetic mutations and histopathomic features. Heatmaps illustrated distinct heterogeneity in tumor and microenvironmental characteristics associated with specific somatic gene alterations across both cohorts (Fig. 8a**, b**). The mutational landscape, stratified by HPSurv, showed that higher risk scores were enriched in patients harboring established driver mutations (Fig. 8c). Notably, mutations in key driver genes, including TP53 (Fig. 8d), CDKN2A (Fig. 8e), and SMAD4 (Fig. 8f) in the PUMCH cohort, as well as ATM (Fig. 8g) in the TCGA cohort, were significantly associated with distinct and histopathomic features of HPSurv (all BH-adjusted *P* < 0.05). The observed biological concordance between image-derived features and well-established oncogenic mechanisms suggests that HPSurv may reflect known cancer biology.

## Discussion

This study developed an AI system (HPSurv) for OS prediction with enhanced reliability for PDAC patients who underwent curative-intent pancreatic surgery. The HPSurv includes tissue classification (PAPF-Net), quantifying histopathological characteristics (histopathomic features), and prognosis prediction (histopathomics model). A retrospective cohort spanning multiple centers, regions, and populations was constructed for external testing. HPSurv demonstrated robust performance for both regression risk prediction and time-dependent event prediction. Furthermore, HPSurv relied solely on postoperative hematoxylin and eosin (H&E) whole slide images commonly used in diagnosis without any extra biological detection, thereby underscoring its clinical applicability. The study adhered to the AI-based biomarker development guidelines to enhance the standardization of the research report^37,38^.

For patients with resectable or borderline resectable cases, the standard line of treatment comprising surgical strategy in conjunction with postoperative chemotherapy remains the main clinical approach for reducing recurrence and metastasis of pancreatic cancer^3^. Clinically, adjuvant treatment regimens encompass monotherapy (gemcitabine), combination chemotherapy (gemcitabine combined with 5-fluorouracil (5-FU), or gemcitabine combined with oxaliplatin) and quadruple therapy (mFOLFIRINOX)^39–41^. Poly (ADP-ribose) polymerase inhibitors, KRAS-targeted therapies, and immunotherapies are also promising options^5^. HPSurv demonstrated robust prognostic performance across multicenter cohorts, supporting its potential value for risk stratification and personalized patient management.

In the study, the HPSurv ensured that tumor spatial heterogeneity was no longer relegated to an abstract concept or a qualitative description. Histopathological features with potential relevance to PDAC prognosis, including tumor proportion, stromal area, tumor–stroma ratio, and the abundance of tumor-infiltrating lymphocytes, were quantitatively characterized and extracted^25,42^. For instance, the morphological size of the stroma is known to be significantly associated with prognostic risk^19^. Similarly, the proportion of nerve involvement is closely linked to the extent of tumor infiltration and metastatic potential, thus making it an important prognostic factor^43^. Higher levels of immune infiltration are often associated with poorer prognosis^44^. Molecular-level analyses further support the notion that histopathological features determine the prognosis of pancreatic cancer in patients.

The performance of HPSurv was attributed to incorporate domain-specific medical knowledge into the development and optimization. Prognostic prediction was achieved through a combination of risk regression and time-dependent event classification, enabling alignment with clinically relevant endpoints. Notably, model performance varied across time horizons, with higher accuracy observed in short-term survival prediction. This may reflect the clinical course of PDAC, where early postoperative recurrence and metastasis are more readily detectable^45^. Notably, baseline models achieved meaningful prognostic performance, with the nonlinear model generally showing superior results. These findings also underscore the importance of jointly optimizing feature representation and predictive modeling for robust survival prediction. From an oncological perspective, early postoperative tumor cells remain active and make recurrence as well as metastasis features easier to detect in pathological images, thus enhancing prediction accuracy^25^. However, temporal changes in the tumor microenvironment and cancer cell characteristics can attenuate key recurrence or metastasis features, which in turn compromises the accuracy of prognosis. From methodology, while postoperative pathological image data is stable, its spatiotemporal invariance limits its applicability. AI models analyze instantaneous postoperative pathological tissue images. This poses challenges for longer-term predictions, as the training data increasingly diverges from real-world conditions^46^. The above characteristics also provide the clinical interpretability of HPSurv for safety of clinical applications.

Notably, although our model demonstrated significantly improved overall survival prediction in a multicenter setting and may help inform postoperative treatment decisions, its clinical application should be interpreted cautiously. Specifically, the present work is based on retrospective data and has not been evaluated in prospective studies designed to assess the impact of model-guided decisions on patient outcomes. Moreover, formal interaction analyses were not performed, and we did not systematically adjust for potential confounders that could bias associations between histopathologic features, treatment choices, and survival. Accordingly, the findings should be viewed primarily as prognostic rather than prescriptive, and external validation—including prospective evaluation with appropriate handling of treatment interactions and confounding—will be needed before the tool can be considered for routine clinical use.

This study has several limitations. First, although multicenter cohorts and an international dataset (TCGA) were included, the geographic diversity of the study population remains limited. Further validation in larger and more diverse populations through prospective international multicenter studies is needed to establish generalizability. Second, the multi-omics analyses based on the PUMCH and TCGA-PAAD cohorts were constrained by a relatively limited number of genomically profiled cases. Although the identified pathways and driver mutations were broadly consistent with established biological mechanisms, additional validation in larger cohorts with paired whole-slide imaging and multi-omics data is warranted. Third, while HPSurv demonstrated robust discriminative performance, calibration varied across external cohorts, likely reflecting differences in patient populations and data distributions. Future prospective validation and calibration strategies may further improve the reliability of individualized risk estimation. Finally, although exploratory analyses suggested distinct outcome patterns across HPSurv-defined risk groups and adjuvant chemotherapy status, the retrospective nature of this study precludes conclusions regarding treatment benefit. Prospective studies incorporating formal treatment–biomarker interaction analyses will be required to determine whether HPSurv possesses predictive value for adjuvant chemotherapy benefit.

In conclusion, HPSurv was developed and validated across multiple cohorts for postoperative overall survival prediction in PDAC. The findings highlight that spatial tumor heterogeneity derived from histopathological images is closely associated with patient prognosis. Moreover, histopathomic features not only enable accurate survival prediction but also provide biologically meaningful insights into the molecular characteristics of the disease.

## Methods

### Study design

In this study, a total of 1020 patients with PDAC were involved in development, internal validation, and external testing, based on three cohorts and an open-source dataset. The primary cohort (PC) and validation cohort (VC) were incorporated from Peking Union Medical College Hospital (PUMCH) (Beijing, China) between Jun, 2011 and Oct, 2021, along with a 3-year follow-up period until October 2024. The PC and VC were split based on chronological order. Specifically, the first 80% of patients (by time) were assigned to the PC, and the remaining 20% were assigned to the VC. The external testing cohort #1 (ExTC1) was incorporated from The Fifth People’s Hospital of Hebei Medical University (FHHMU) (Shijiazhuang, China) during July, 2016 to Dec, 2019. ExTC2 was incorporated from the People’s Liberation Army General Hospital (PLAGH) (Beijing, China) between Jan, 2018 and Dec, 2018. ExTC3 was incorporated from the Nanfang Hospital Ganzhou Hospital (NHGH) (Ganzhou, China) during Jan, 2022 to Jan, 2023. The retrospective inclusion criteria for the participants were: 1) unequivocally diagnosed with PDAC by two expert pathologists and 2) available FFPE tissue slides. The exclusion criteria were: 1) pre-operative therapy; 2) censored in six months; 3) image noises impacting pathological assessment.; 4) death within one month; 5) M1 stage; and 6) concurrent malignancies. Based on the above, a cohort of160 patients with PDAC was incorporated from TCGA-PADD between 2001 and 2013 as ExTC4.

Model development was conducted exclusively in the primary cohort (PC), including tissue classification, feature selection, and overall survival regression prediction. The model was subsequently validated using independent external testing cohorts (ExTC1-4) to assess its performance across multiple centers, time periods, regions, and populations. In the study design, all datasets were partitioned at the patient level to minimize data leakage, with model development restricted to the training set and independent evaluation performed on validation and testing sets. In addition, the patch-level classification task was performed using a fully independent dataset with no overlap of patients or slides with the prognostic cohorts. The optimal cut-off was determined in the PC using the Youden index for 24-month mortality and applied unchanged to all validation and testing cohorts (Supplementary Table 16).

Due to the retrospective nature of the study, the ethics committee of the Peking Union Medical College Hospital (approval no. I-24PJ0742) and the First Medical Center of People’s Liberation Army General Hospital (approval no. S2025-517-01) approved the study and waived the requirement for informed consent. Data from additional centers (PLAGH, NHGH) were retrospectively collected and included under the ethical approval of PUMCH, which approved the use of de-identified data from collaborating institutions. This study adhered to the Declaration of Helsinki and obtained approval from the ethics committee. The reporting format of this study follows the guidelines of the Checklist for Artificial Intelligence in Medical Imaging (CLAIM)^47^ and Transparent Reporting of a Multivariable Prediction Model for Individual Prognosis or Diagnosis (TRIPOD) guidelines^37^.

### Whole slide image preparation and pathologist-level annotation

The histopathological images were derived from paraffin-embedded postoperative tissue sections of patients with PDAC, which were stained with H&E. Pathology diagnostic slides were selected for analysis, and all images underwent a quality assessment performed by pathologists. This assessment evaluated the presence of tissue damage, contamination, blurring, noise, and staining artifacts. Furthermore, computer scientists were invited to evaluate image quality from the perspective of digital image analysis requirements. Low-quality samples were excluded from the dataset. Slides from PUMCH were scanned using a HAMAMATSU Nanozoomer S360 digital scanner (40×, 0.23 µm/pixel) and a TEKSQRAY SQS-40P digital scanner (20×, 0.19 µm/pixel). Similarly, slides from PLAGH were scanned using a UNIC PRECICE 500B digital scanner (40×, 0.25 µm/pixel). Slides from FHHMU and NHGH were scanned using a KFBio KF-SCAN-PL digital scanner (40×, 0.25 µm/pixel).

The PDAC exhibits pronounced desmoplasia, diffusely distributed tumor ducts, and a complex microenvironment, making exhaustive whole-slide annotation challenging and prone to bias. Therefore, a tailored annotation strategy was adopted, with more comprehensive labeling for small but highly heterogeneous regions (e.g., invasive carcinoma) and selective annotation for larger, more homogeneous regions (e.g., normal tissue), enabling efficient annotation while capturing intratumoral heterogeneity (Fig. 2a). To enhance representativeness, each whole-slide image was obtained from a different patient. Annotation quality and interobserver consistency were ensured through a multi-step review process, including independent assessments by two experienced pathologists and quantification using the intraclass correlation coefficient (ICC). Only annotations with ICC > 0.95 were included, while discrepant cases were resolved through consensus review by a senior pathologist. The panel labeled 12 distinct categories of pancreatic tumor pathology: cancer or high-grade pancreatic intraepithelial neoplasia (Ca&HPIN), low-grade pancreatic intraepithelial neoplasia (LPIN), normal tissue, acinar atrophy or catheterization (Aa&C), tumor stroma (TUMST), immune infiltration (IMMI), lymph node (LYN), nerve (NERV), blood vessel (BV), blood cell aggregation (BCA), necrosis (NEC), and others (OTH).

In total, 101 H&E slides of human cancer tissue were annotated by pathologists from PUMCH, resulting in 840,000 image patches of size 256 × 256 pixels at a resolution of 0.5 μm/pixel, named PUMCH-PDAC-HE. Of these, patches from 94 slides were allocated for transfer learning and classifier training, whereas the remaining 10% patches of seven slides were reserved for validating classification performance independently. To prevent the performance evaluation bias caused by uneven tissue distribution, twelve categories were assigned according to the minimum amount of tissue in the verification.

### Transfer learning of pathology foundational model for pathological tissue classification in whole slide image

The pathology foundation model was used as a pathology image patch feature extractor. The base model UNI^32^ was selected as the pre-training parameters and structure, owing to its advantages in the patch-level classification of pancreatic cancer pathological tissue (Supplementary Table 3). For better performance, the classification model is not only required to pay attention to cellular details at high magnification, but should also possess environmental perception combined with a wide field of view. Therefore, a pyramid attention-based pathology foundation model transfer learning network (PAPF-Net) was proposed to simultaneously input pixels at 20× (0.5 µm/pixel), 10× (1 µm/pixel), and 5× (2 µm/pixel) magnification. Additionally, the attention block was adopted to fuse feature information from different magnifications of the same target anchor with multiple views. Multi-head attention was used to output the likelihood of the variant tissues. In the transfer learning phase, the cross-entropy function was used to measure the multi-classification error between the predicted results and the true labels.

1 $${Attention}\left(Q,K,V\right)={softmax}\,\left(\frac{Q{K}^{{\rm{\top }}}}{\sqrt{{d}_{{head}}}}\right)$$

Wherein, Q, K, and V represented the query, key, and value, respectively. These were derived from the encoding of the feature. Additionally, ${d}_{{head}}$ indicated the dimensionality of each attention head.

2 $${MultiHead}\left(Q,K,V\right)={Concat}\left({{head}}_{1},\,{{head}}_{2},\ldots ,{{head}}_{h}\right)* {W}^{O}$$

Wherein, ${W}^{O}$ denoted a Multilayer Perceptron for output.

The training samples were selected using an 80% overlapping sliding window strategy based on expert labeling to maximize the diversity and richness of the training dataset. To meet the rectangular input size requirement, irregular samples with less than 50% of valid ROI (Region of Interest) labeled pixels in the target domain were excluded to mitigate the potential negative impact of weak supervision. Each patch was assigned a label consistent with the corresponding annotated category.

Furthermore, to ensure a diverse training dataset, all segmented patches were evaluated using feature-based structural similarity and overly similar training blocks were removed to prevent overfitting. The issue of class imbalance was addressed by balancing the dataset following the method of under-sampling or over-sampling samples from twelve categories based on their mean distribution.

Color normalization was performed to enhance sample consistency and minimize interference caused by variations in scanning devices during the modeling process. Additionally, standard data augmentation techniques were employed to improve model generalization. These techniques included image transformations such as flipping, rotation, affine transformations, and adding random Gaussian noise. More details of modeling were introduced in Supplementary Table 18.

### Construction of histopathological map for pancreatic ductal adenocarcinoma

The trained PAPF-Net was used for end-to-end classification of the tissue in foreground area of WSI, by sliding without overlapping. The likelihood estimates for the twelve categories were simultaneously provided in the output. Additionally, the categories of the target tiles were determined by the softmax function of predictions, which yielded the highest score. The histopathological map of PDAC (Histomap) was constructed via scanning and classifications. Furthermore, the combination of thresholding and Otsu’s method was used to segment the foreground in WSI, which improved computational efficiency. Notably, the background, holes, and broken micro-tissues were excluded. The multi-magnification images in the test were derived from a combination of images of the same size centered on the highest magnification tiles, including the 20×, 10×, and 5× images. The resolutions of the histomap provided a basis for calculating histopathological features with low information redundancy, which focus on tumor spatial heterogeneity.

### Quantification of histopathological heterogeneity based on histopathomic features

Tumor heterogeneity includes intra- and inter-tissue heterogeneity. To quantify the spatial heterogeneity of PDAC tissue, histopathomic features were extracted from whole-slide images (WSIs) using the PyHistopathomics Toolkit. Histopathomic features were derived from different types of tissue and comprised statistical features, morphological features, as well as textural features (Supplementary Table 19). The statistical features consisted of tissue area, absolute proportion of pathological tissue, relative proportion of pathological tissue categories, typicality ratio, typicality, typicality ratio between tissues, and Shannon entropy. Similarly, the morphological features comprised centroid, perimeter, ellipse similarity, minimum closed circle similarity, and convexity. Gray-level co-occurrence matrix (GLCM) and Local Binary Pattern (LBP) were used to extracted texture features. The textural features included quantized variant tissues of contrast, homogeneity, stability and heterogeneity, and were evaluated at resolutions of 16 μm/pixel and 32 μm/pixel, which was designed for differentiating between dense type, loose type, inflammatory type, immune rejection type, and immune desert type tissue features. For slides containing multiple unconnected tissue regions, summary statistics (mean, variance, maximum, and minimum) were computed and used as feature representations for the corresponding tissue categories. The histomap was used to distinguish different tissue regions from WSI.

### Features screening for overall survival

The extracted histopathomic features contained 2742 entries. To reduce the information redundancy in the modeling process, features related to PDAC prognosis were screened through a coarse-to-fine strategy. Time-dependent significance was used to identify features of death occurring within fixed time periods using the Mann–Whitney U test for the selected feature pooling, comprising 3-, 6-, 12-, 18-, 24-, and 36-month periods. Features with a *p*-value less than 0.05 were filtered as those significantly related to overall survival, and features that were repeatedly selected were subsequently merged. Linear correlations between features were assessed using the Pearson test, whereby features with value of R greater than 0.95 were excluded. Thereafter, the Least Absolute Shrinkage and Selection Operator (LASSO) Cox regression was applied to the feature selection. To improve follow-up data utilization, overall survival was right-truncated according to the observation time; this was consistent with the strategy based on time-dependent significance. The feature weights were confirmed by five-fold cross-validation, and features with a weight value of 0 were excluded. A feature weight of 0 indicates that the feature can neither promote nor inhibit the disease and is irrelevant to OS prediction. Following the feature screening, the retained features were used for modeling.

### Histopathomics model and overall survival prediction of pancreatic ductal adenocarcinoma

A transformer survival network was built for OS prediction, named histopathomics model (Supplementary Fig. 12). The input of histopathomics model comprised the thirty-nine histopathomic features, whereas the output was the prediction of risk of death. The architecture of this system was based on the attention unit of transformers to capture attention among features for risk prediction. For training, the survival composite loss ${l}_{{Surv}}$ aimed to minimize both the proportional hazards regression loss ${l}_{{regression}}$ and time-dependent event classification loss ${l}_{{event}}$.

3 $${l}_{{Surv}}=\alpha * {l}_{{regression}}+(1-\alpha )* {l}_{{event}}={L}_{{Surv}}\left(p,{tp},E,T\right)$$

Wherein, p represented the vector of predicted results, tp represents timepoints of events, E represented the true event indicator vector, T indicated the vector of actual survival time. In the loss function, the weight of α was used to personalize the prediction task to favor quantitative prediction of risk differences between individuals or qualitative prediction of survival events.

The ${l}_{{regression}}$ was calculated by DeepSurv loss derived from CPH model.

4 $${l}_{{regression}}=-\sum _{i:{Ei}=1}\left(h\theta \left({xi}\right)-\log \sum _{j\in {\mathcal{R}}\left({T}_{i}\right)}\exp (h\theta \left({xi}\right))\right)$$

where ${x}_{i}$ denotes the feature vector of the i-th patient, ${h}_{\theta }({x}_{i})$ represents the predicted log-risk score output by the neural network with parameters θ, ${T}_{i}$ is the observed survival time, and ${E}_{i}\in \{\mathrm{0,1}\}$ is the event indicator (1 for event occurrence and 0 for censoring). The risk set ${\mathcal{R}}{{(}}{T}_{i})$ includes all individuals who are still at risk at time ${T}_{i}$(i.e., those with ${T}_{j}\ge {T}_{i}$).

The ${l}_{{event}}$ was evaluated by binary cross-entropy (BCE) of every time-dependent event.

5 $${l}_{{event}}=\sum _{{tp}=[{6,12,24,36}]}-\frac{1}{N}\mathop{\sum }\limits_{i=1}^{N}\left[{y}_{i}\log ({p}_{i})+(1-{y}_{i})\log (1-{p}_{i})\right]$$

where N denotes the number of samples, ${y}_{i}\in \{\mathrm{0,1}\}$ represents the ground truth label of the i-th sample, and ${p}_{i}\in [\mathrm{0,1}]$ is the predicted probability of the positive class.

### Transcriptomic analysis and genomic analysis

Genomic and transcriptomic analyses were conducted on a subset of the PUMCH cohort (*N* = 82) with available targeted next-generation sequencing (NGS) data. Due to the high cost of clinical NGS and the limited therapeutic options for PDAC, this subset represents all patients in the PUMCH cohort with available genomic profiles. For external validation and broader transcriptomic analysis, data from the TCGA-PAAD cohort (*N* = 127) were utilized.

Differential gene expressions between high- and low-risk groups, as defined by the HPSurv, were evaluated using the R package DESeq2. To characterize the biological processes associated with histopathomic features of HPSurv, enrichment analyses of KEGG pathways and Gene Ontology (GO) terms were performed using the R package clusterProfiler. To ensure statistical rigor, multiple testing correction was applied using the Benjamini-Hochberg (BH) method, and only pathways or genes with a false discovery rate (FDR)-adjusted *P* value < 0.05 were considered statistically significant. A protein–protein interaction (PPI) network was subsequently constructed using the STRING database and visualized in Cytoscape to identify key functional gene modules.

Somatic mutations from both the PUMCH (*N* = 82) and TCGA-PAAD (*N* = 127) cohorts were analyzed to assess the association between the mutational landscape and histopathomic features. The mutational status of key PDAC driver genes (e.g., TP53, CDKN2A, SMAD4, and ATM) was compared with histopathological characteristics using Wilcoxon rank-sum tests. These analyses were designed to demonstrate that the image-based model captures established oncogenic mechanisms.

#### Statistical analysis

The C-index was employed in multicenter testing to assess the overall performance of regression prediction of OS. Additionally, the AUC, sensitivity (SEN), specificity (SPE), PPV, and NPV were employed to evaluate classification performance for time-dependent event (mortality) predictions. The 95% confidence interval (CI) was calculated for these performance measures using bootstrapping (1000 bootstrap intervals). Furthermore, Kaplan–Meier analysis and log-rank tests were applied to assess the OS outcomes among patients and subgroups with clinical factors. Cox proportional hazards (CPH) analysis was used to calculate hazard ratios with 95% Cis, to confirm the prognostic values of distinct variables. The time-dependent analysis was implemented based on time-dependent ROC, decision curve, and calibration curve for 6-month, 12-month, 18-month, 24-month, and 36-month mortality observations.

Quantitative statistics were presented as mean (SD) or median (IQR). Continuous variables were compared using Student’s t-test or Wilcoxon signed-rank tests, whereas categorical variables were compared using the *χ*² test or Fisher’s exact test. All statistical analyses were two-sided, and p-values of less than 0.05 indicated statistical significance.

### Computational hardware and software

The framework was implemented on a workstation equipped with an NVIDIA GeForce RTX 4090 (24 GB) GPU, an Intel i9-13900 CPU, and 32 GB RAM. Model training and inference were performed using a single RTX 4090 GPU. The software environment was based on Python 3.10.13, with deep learning models implemented in PyTorch 2.0.1 (CUDA 11.7). Statistical analyses were conducted using R (version 4.4.0; packages pROC and PredictABEL) and Python (version 3.10.13; packages scikit-learn 1.2.1 and scikit-survival 0.21.0). Data visualisation was performed using the seaborn package (version 0.13.0) in Python.

## Supplementary information

Supplemental Information.

## Data Availability

The TCGA dataset, including whole-slide images and survival information, is publicly available through the NIH Genomic Data Commons (https://portal.gdc.cancer.gov/). Whole-slide images from PUMCH, FHHMU, PLAGH, and NHGH are not publicly available due to ongoing institutional ethical and legal considerations. De-identified data, including whole-slide images and associated clinical information, are available from the corresponding authors upon reasonable request. Requests for data access should be directed to liangzy@pumch.cn and will be evaluated by the authors in accordance with institutional policies. Data will be shared for academic research purposes only and subject to a data use agreement and, where applicable, institutional review board approval. All data necessary to support the findings of this study are included in the article and its Supplementary Information.

## Code availability

The underlying code for this study is available on GitHub and can be accessed via this link [https://github.com/StandWisdom/PyHistoPahtomics-Toolkit].
