# Supplementary material for: AI-driven tumor heterogeneity quantification and survival prediction in pancreatic ductal adenocarcinoma
Source: NPJ Digit Med. 2026 Jul 20;9:881. doi: 10.1038/s41746-026-02979-7 (PMC13751621; doi:10.1038/s41746-026-02979-7)
Supplement: Supplementary file 1 [file 41746_2026_2979_MOESM1_ESM.pdf]

Supplementary Information

Supplementary Figures

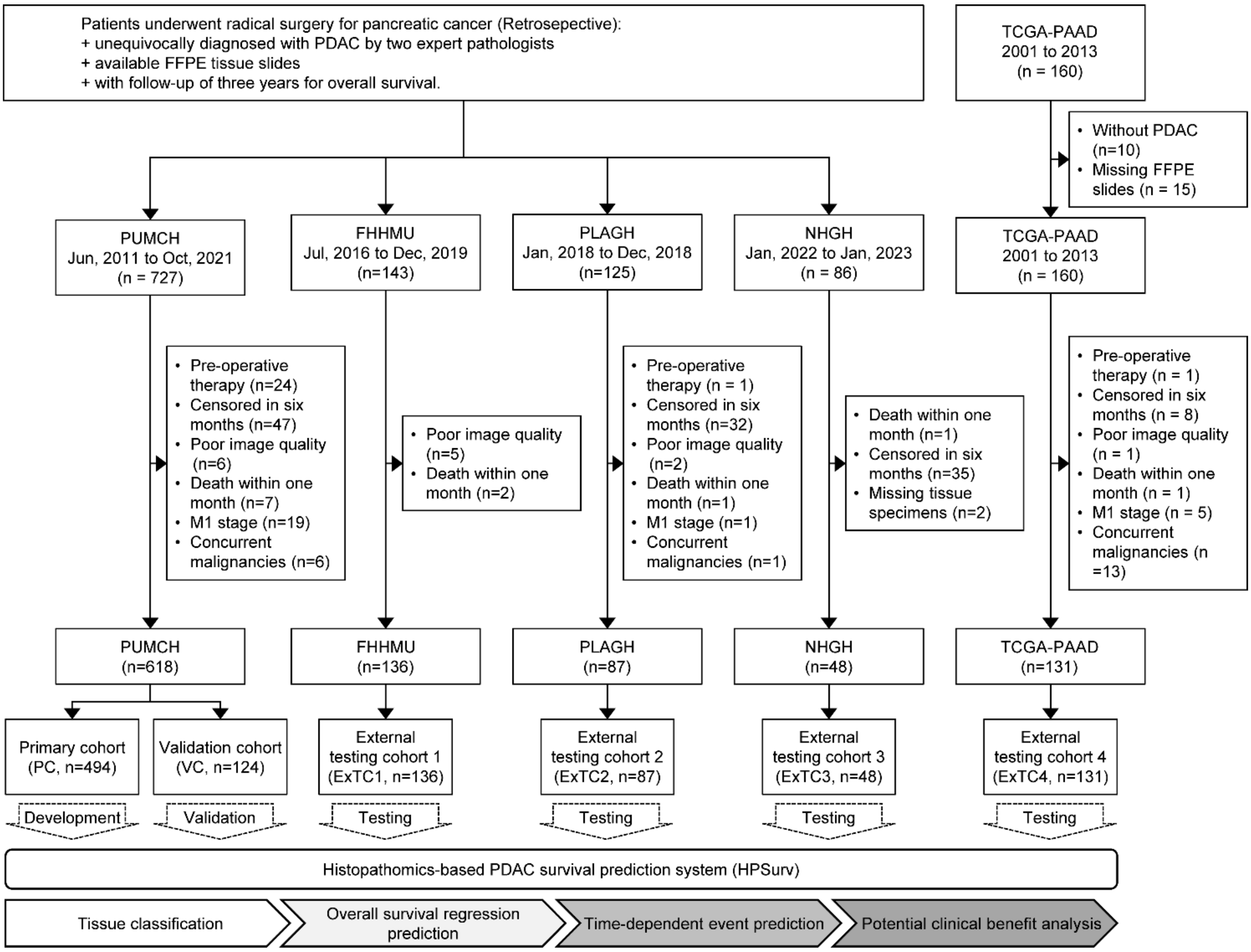

Supplementary Fig.1. Inclusion and exclusion criteria for construction of a multicenter clinical cohort. The primary cohort (PC) and validation cohort (VC) derived from the PUMCH dataset were split based on chronological order. Model development is independent of validation and external multi-center testing.

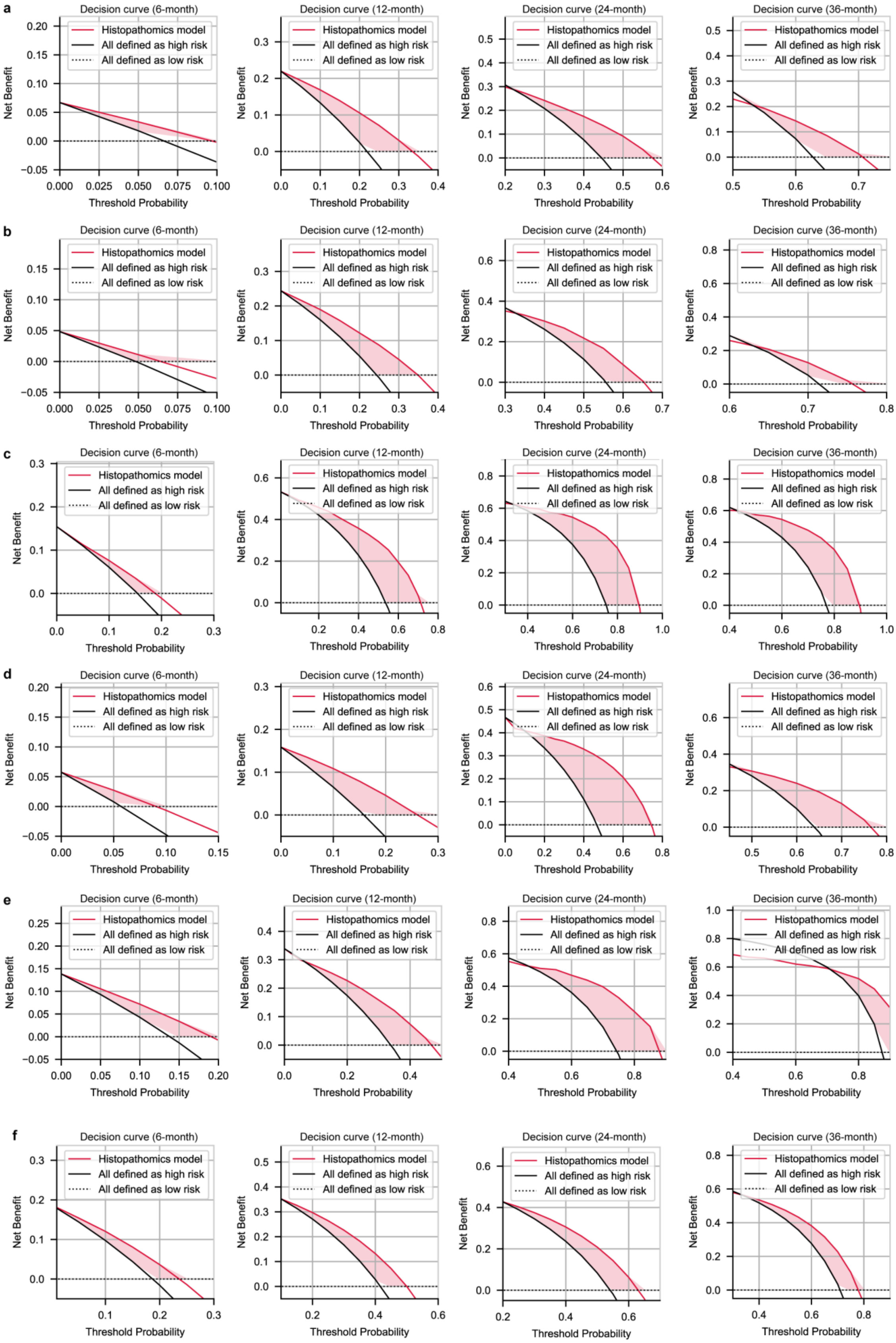

Supplementary Fig.2. Decision curve analysis of time-dependent event (death) prediction based on HPSurv in developing and multicenter testing. (a) Primary cohort. (b) Validation cohort. (c) External testing cohort 1. (d) External testing cohort 2. (e) External testing cohort 3. (f) External testing cohort 4.

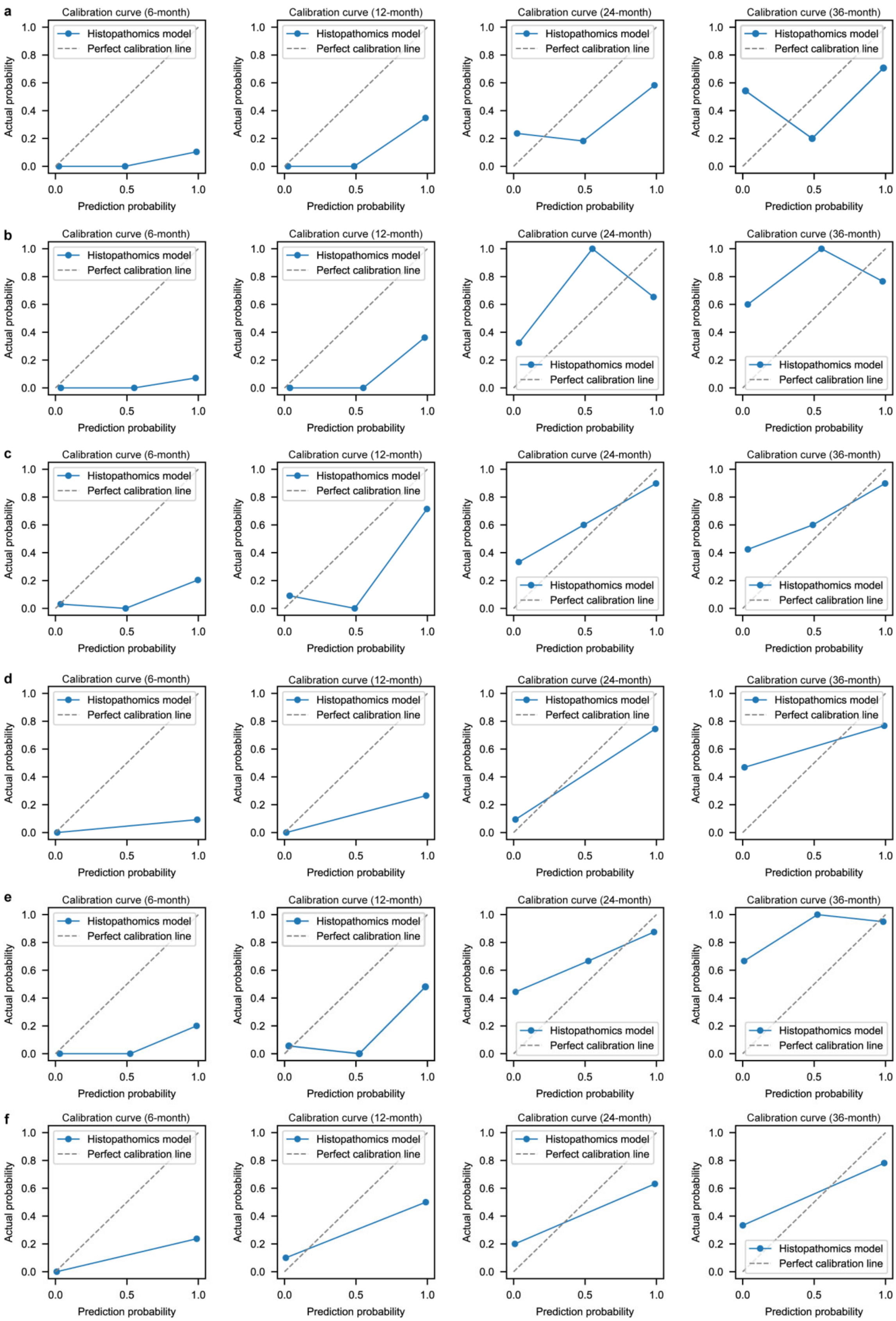

Supplementary Fig.3: Calibration curve analysis of time-dependent event (death) prediction based on HPSurv in developing and multicenter testing. (a) Primary cohort. (b) Validation cohort. (c) External testing cohort 1. (d) External testing cohort 2. (e) External testing cohort 3. (f) External testing cohort 4.

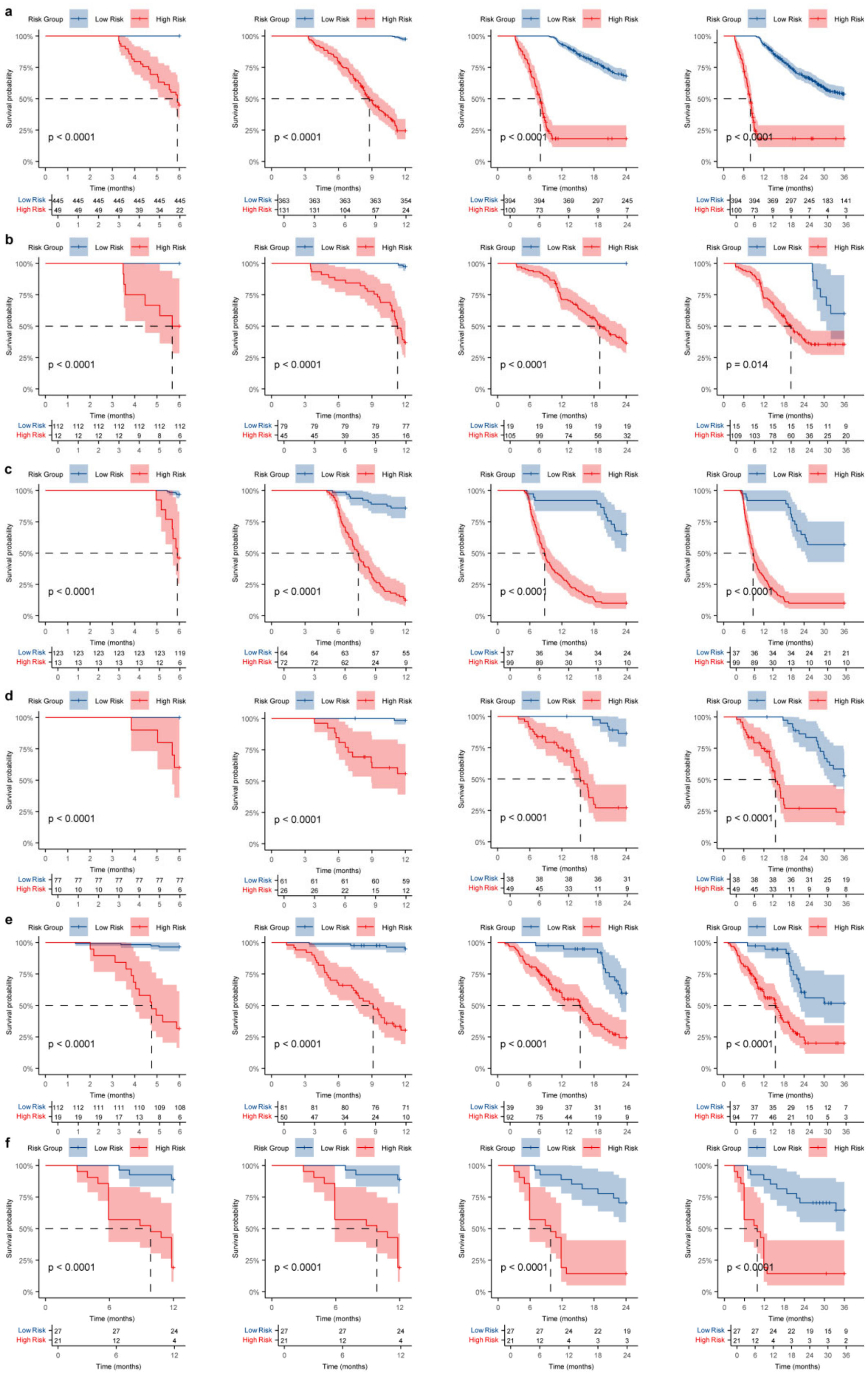

Supplementary Fig.4. Kaplan-Meier survival curve analysis of overall survival (OS) risk regression prediction based HPSurv in developing and multicenter testing. Different cut-off times were used to evaluate the performance and clinical applicability of the model. (a) Primary cohort. (b) Validation cohort. (c) External testing cohort 1. (d) External testing cohort 2. (e) External testing cohort 3. (f) External testing cohort 4.

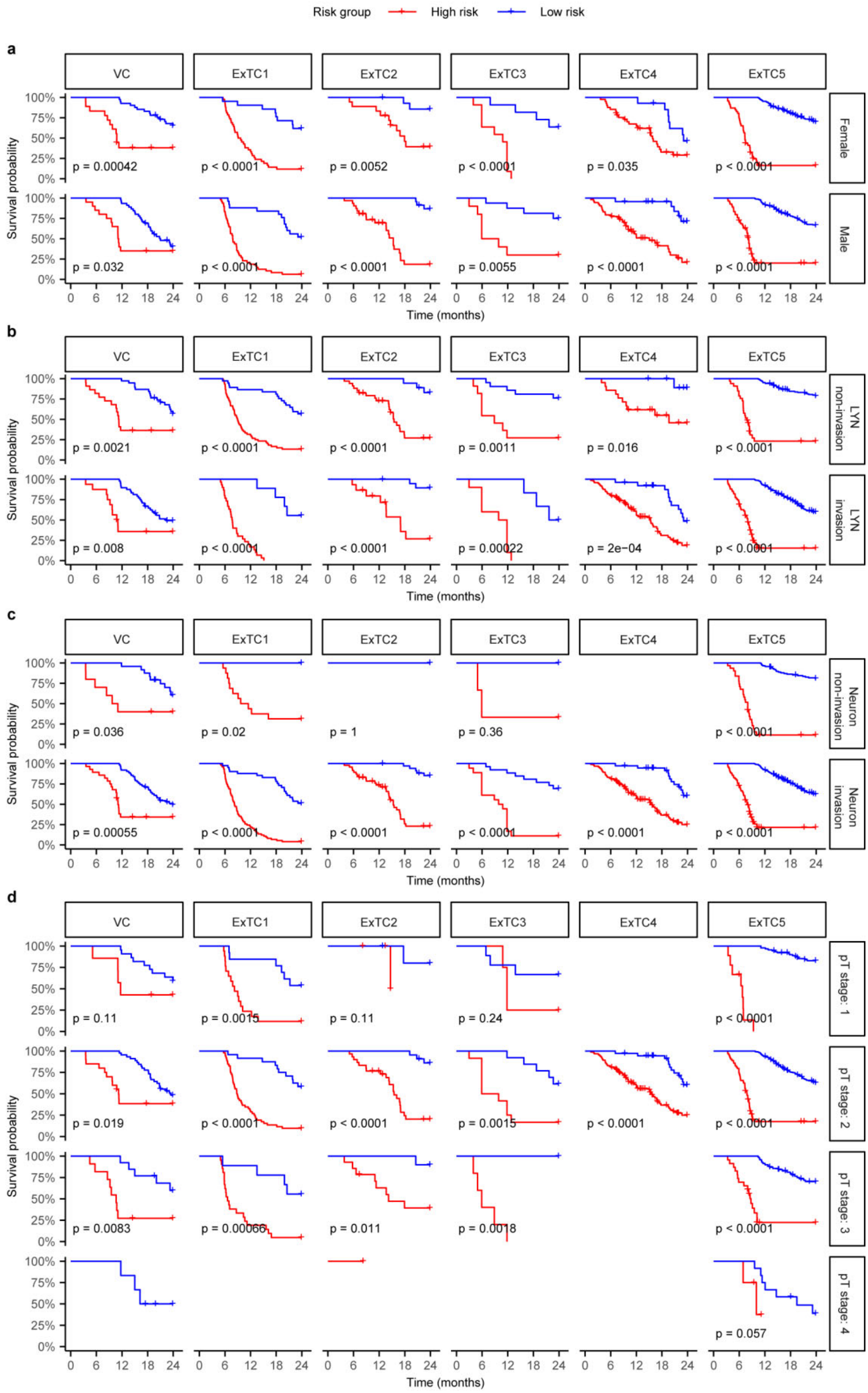

Supplementary Fig.5. Kaplan-Meier survival curve analysis of overall survival (OS) risk regression prediction based HPSurv in clinical subgroups. (a) Gender. (b) Lymph node (LYN) invasion. (c) Neuron invasion. (d) pathological T stage. PC, primary cohort; VC, validation cohort; ExTC1, external testing cohort 1; ExTC2, external testing cohort 2; ExTC3, external testing cohort 3; ExTC4, external testing cohort 4.

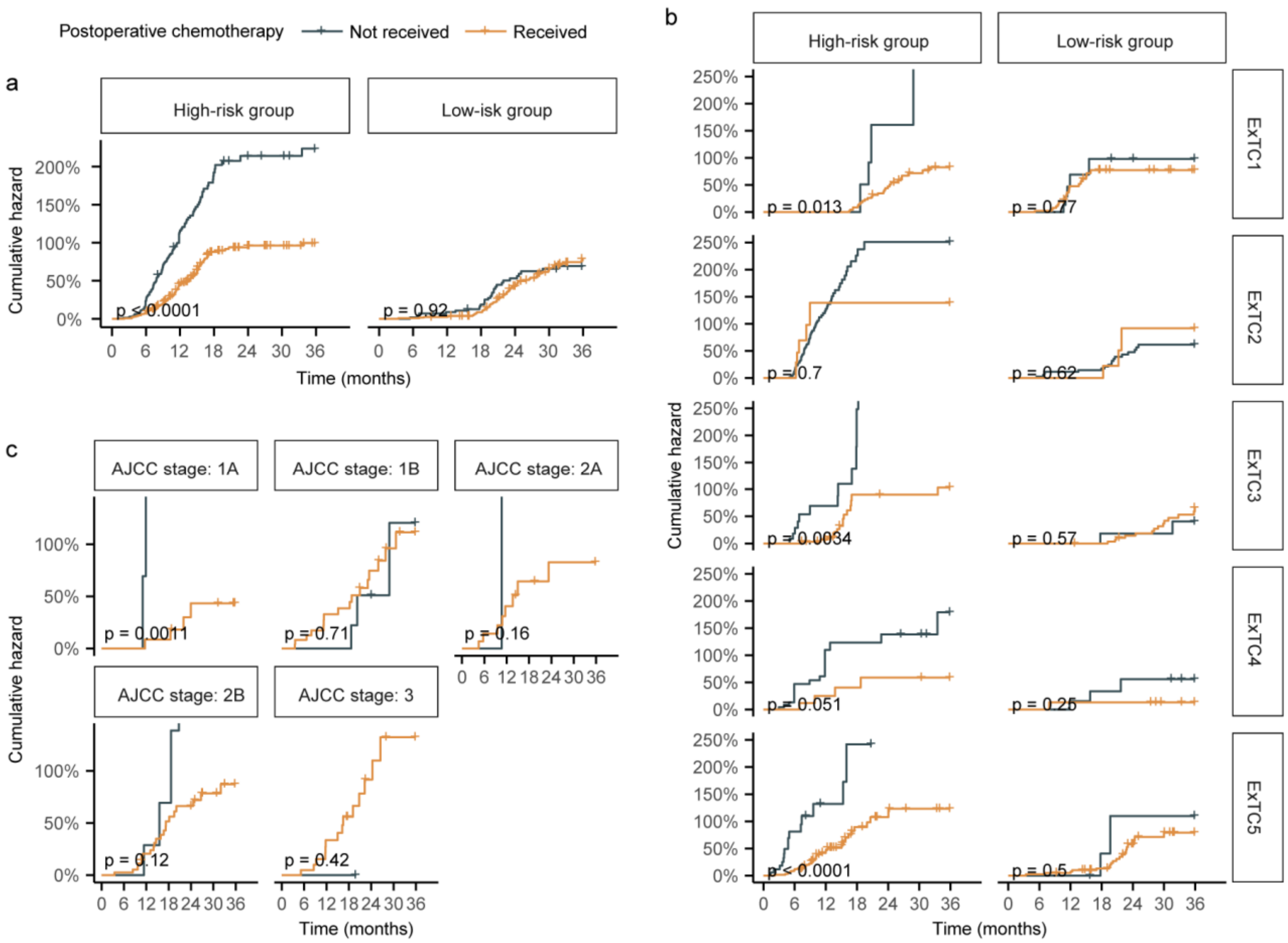

Supplementary Fig.6. Stratified analysis based on HPSurv for postoperative adjuvant chemotherapy (ACT) (Single-drug or double-drug combination chemotherapy). (a) Overall comparison between high-risk group and low-risk group based on HPSurv. (b) Comparison between high-risk group and low-risk group based on HPSurv in multiple centers. (c) Overall comparison between high-risk group and low-risk group based on HPSurv in subgroups of American Joint Committee on Cancer (AJCC) stage.

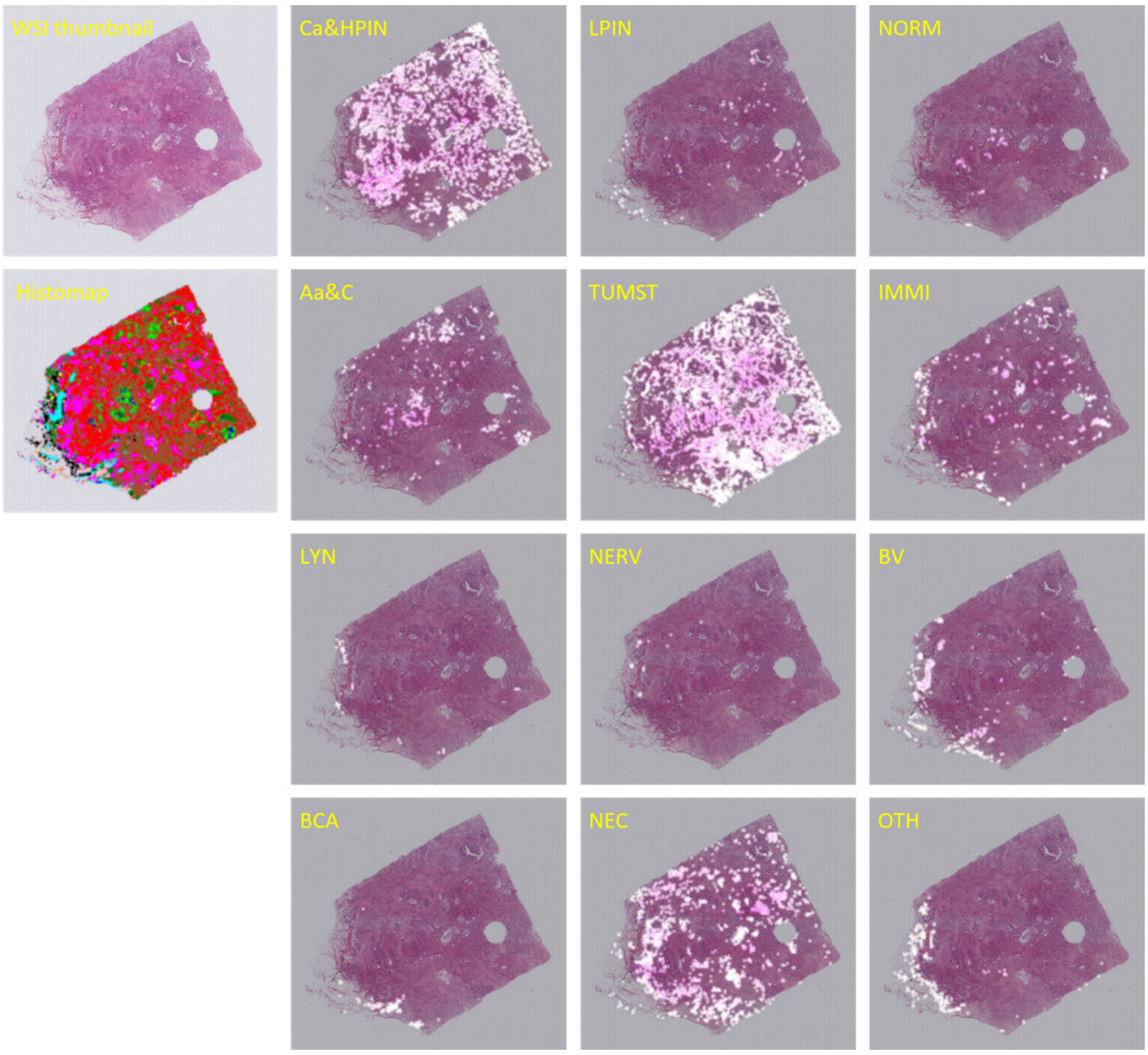

Supplementary Fig.7. Attentions for quantitative extraction of histopathomic features across different pathological tissue types. Ca&HPIN, cancer or high-grade pancreatic intraepithelial neoplasia; LPIN, low-grade pancreatic intraepithelial neoplasia; Aa&C, normal tissue; acinar atrophy or catheterization; TUMST, tumor stroma; IMMI, immune infiltration; LYN, lymph node; NERV, nerve; BV, blood vessel; BCA, blood cell aggregation; NEC, necrosis; OTH, others.

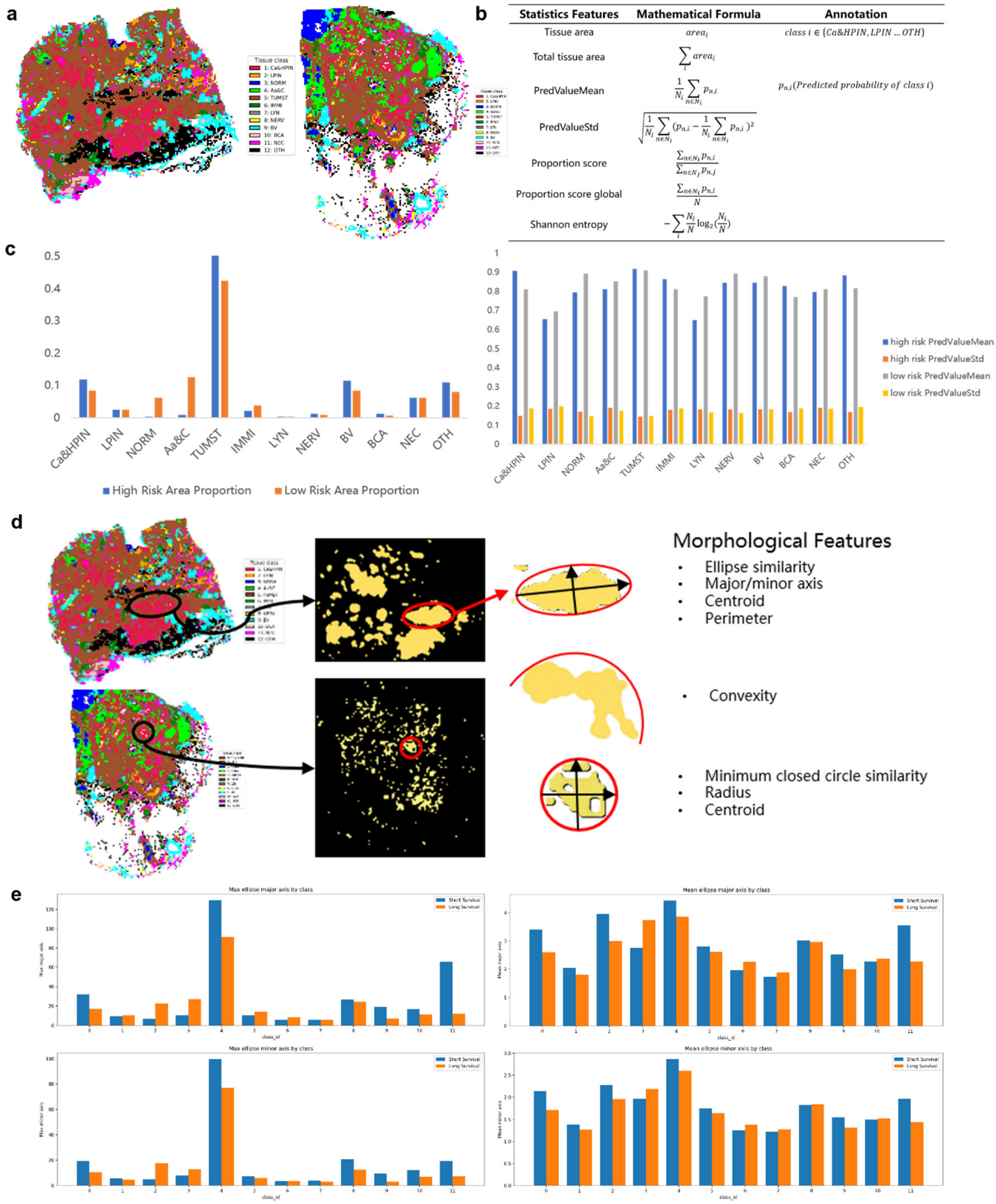

Supplementary Fig.8. Visualization of statistical and morphological histopathomic features derived from Histomaps.(a–c) Representative visualization and distribution of statistical histopathomic features, including tissue area proportions and class-specific prediction-value summaries in high-risk and low-risk regions. (d,e) Representative visualization and distribution of morphological histopathomic features, including ellipse-based shape descriptors across pathological tissue classes in short-survival and long-survival groups.

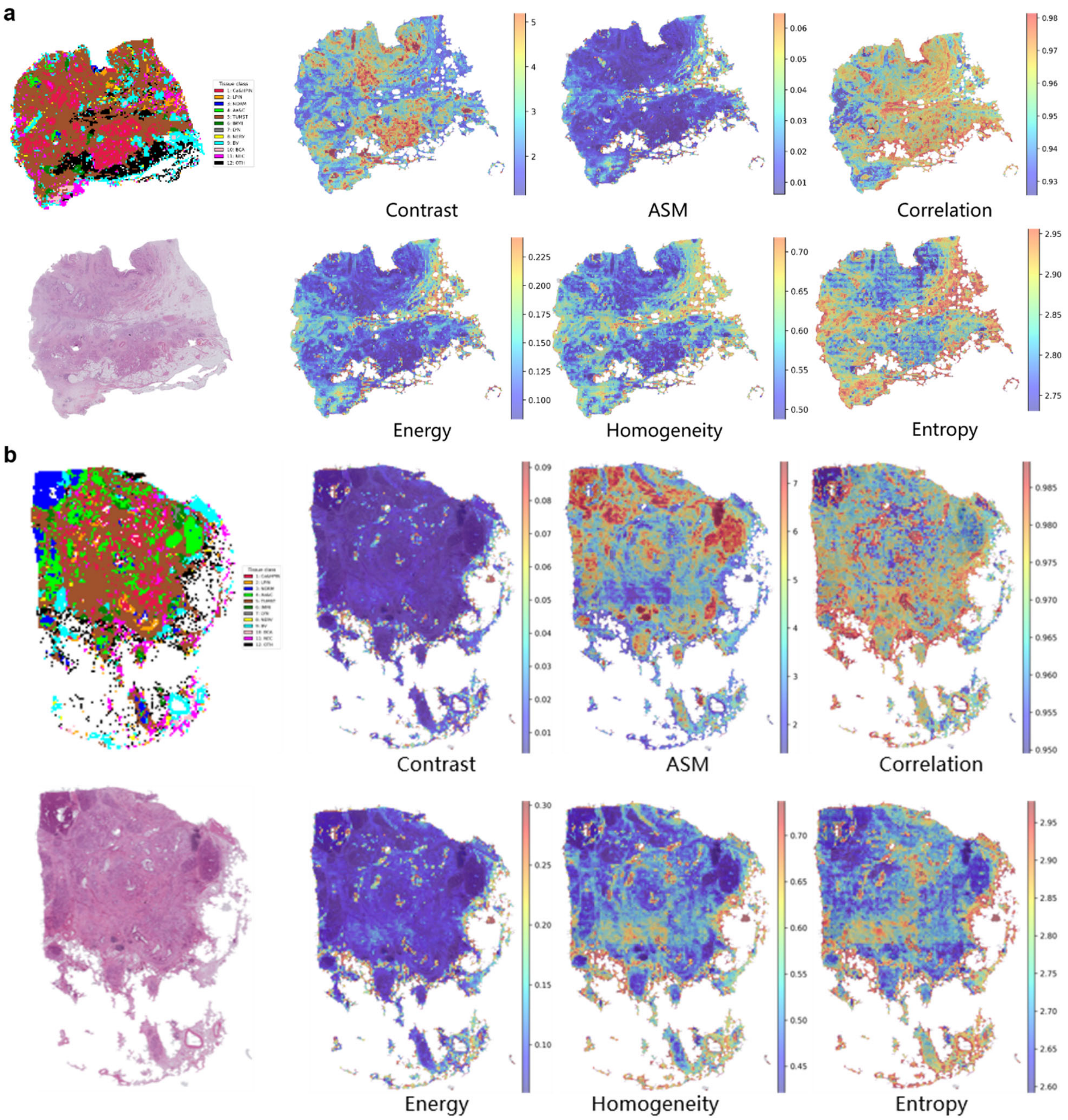

Supplementary Fig.9. Visualization of texture histopathomic features derived from representative PDAC whole-slide images. Representative H&E images and corresponding Histomaps are shown alongside spatial heatmaps of selected texture histopathomic features. The heatmaps illustrate the regional distribution and heterogeneity of quantitative tissue texture features across different pathological tissue regions.

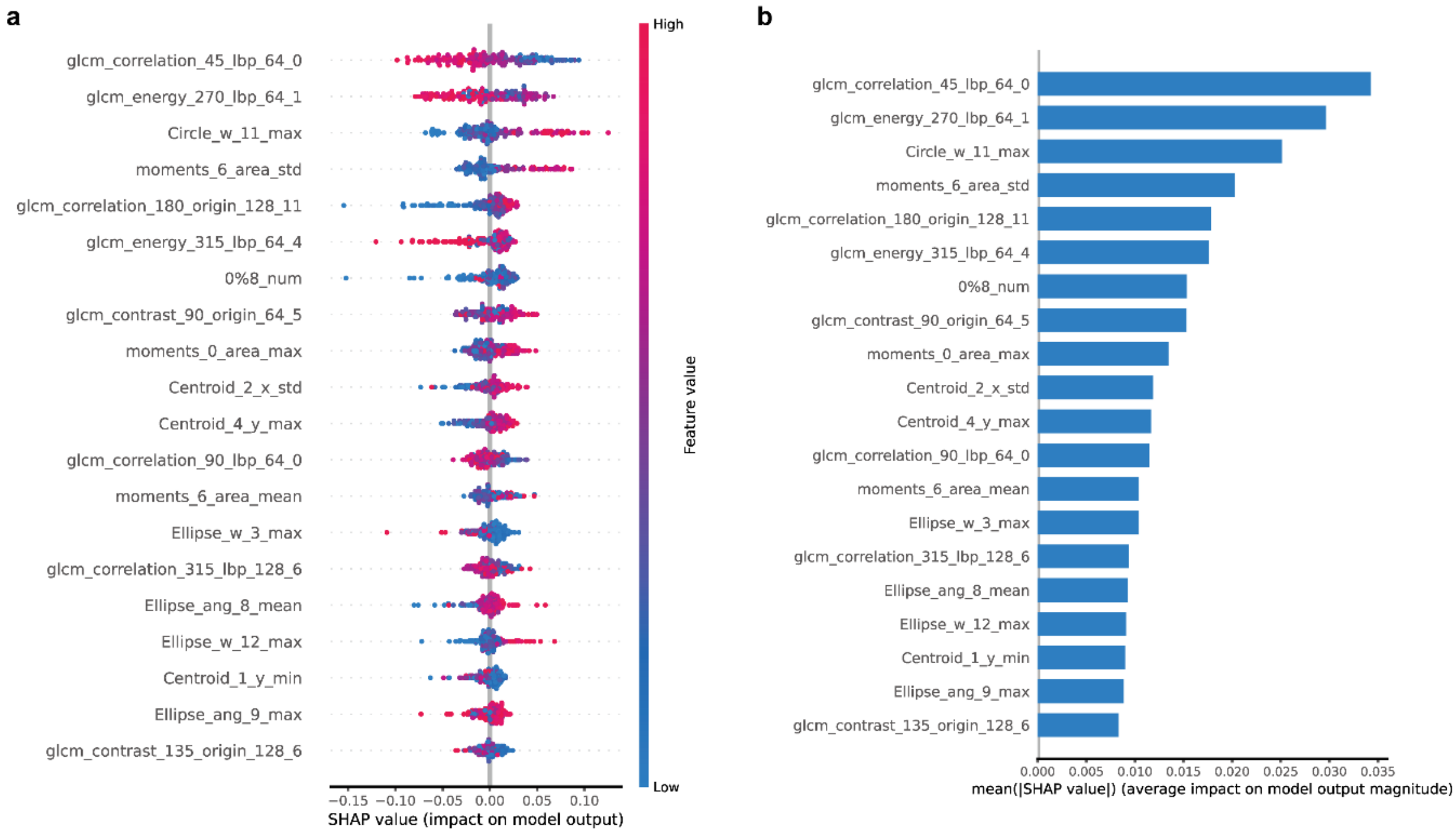

Supplementary Fig.10. SHapley Additive exPlanations (SHAP) Analysis of Feature Importance for HPSurv. (a) Feature Importance Summary. (b) Bar plot based on mean SHAP values of histopathomic features in modeling.

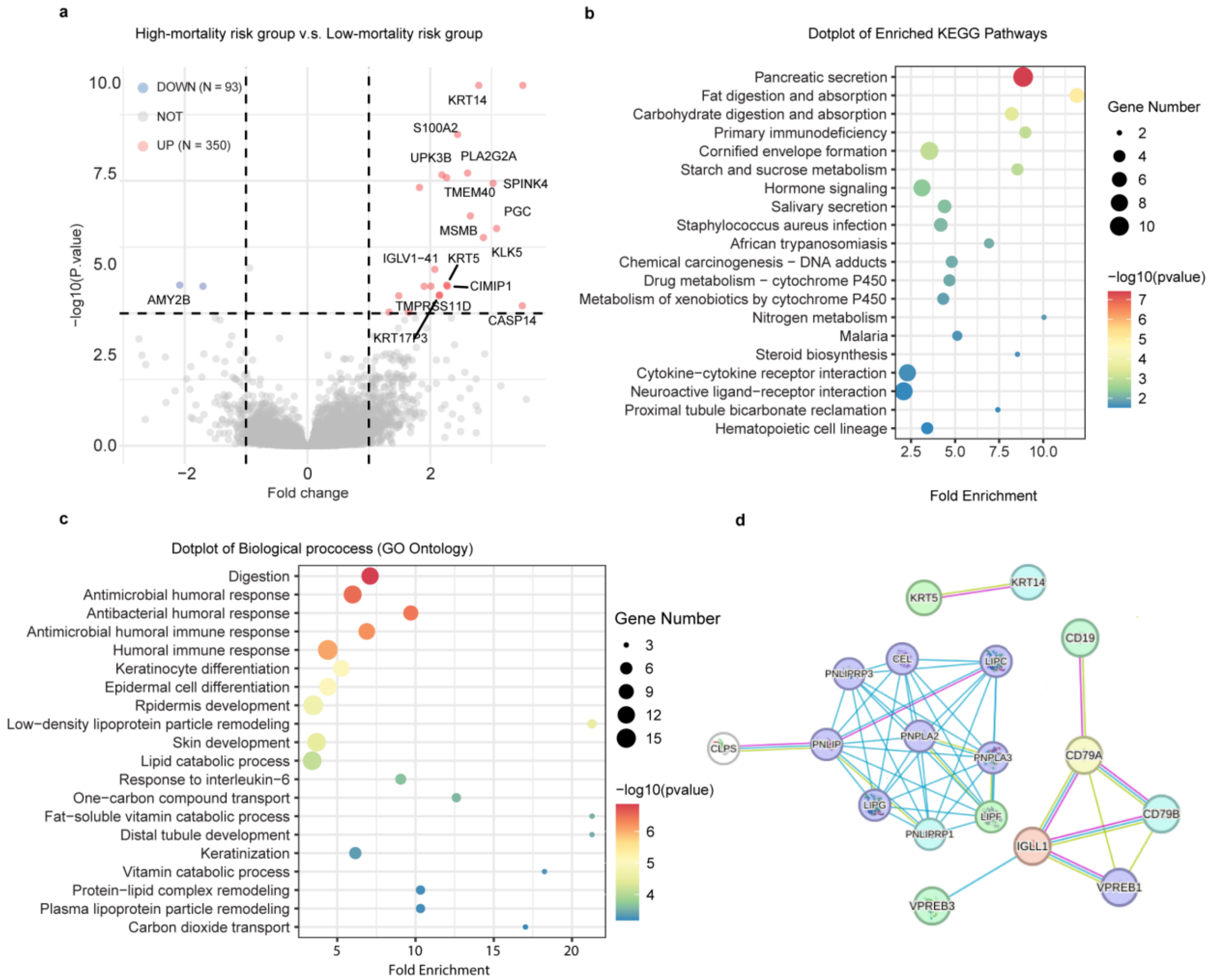

Supplementary Fig.11. Differential gene expression and functional enrichment analyses based on HPSurv. (a) Volcano plot shows that 350 genes were shown to be up-regulated in the high-mortality group, and 93 genes are shown to be down-regulated in the high-mortality group. (b-c) Dotplot shows that several KEGG pathways (b) and GO biological processes (c) are differentially enriched in high-mortality and low-mortality risk groups. (d) Protein-protein interaction (PPI) network analysis reveals the regulatory network of differential-expressed genes.

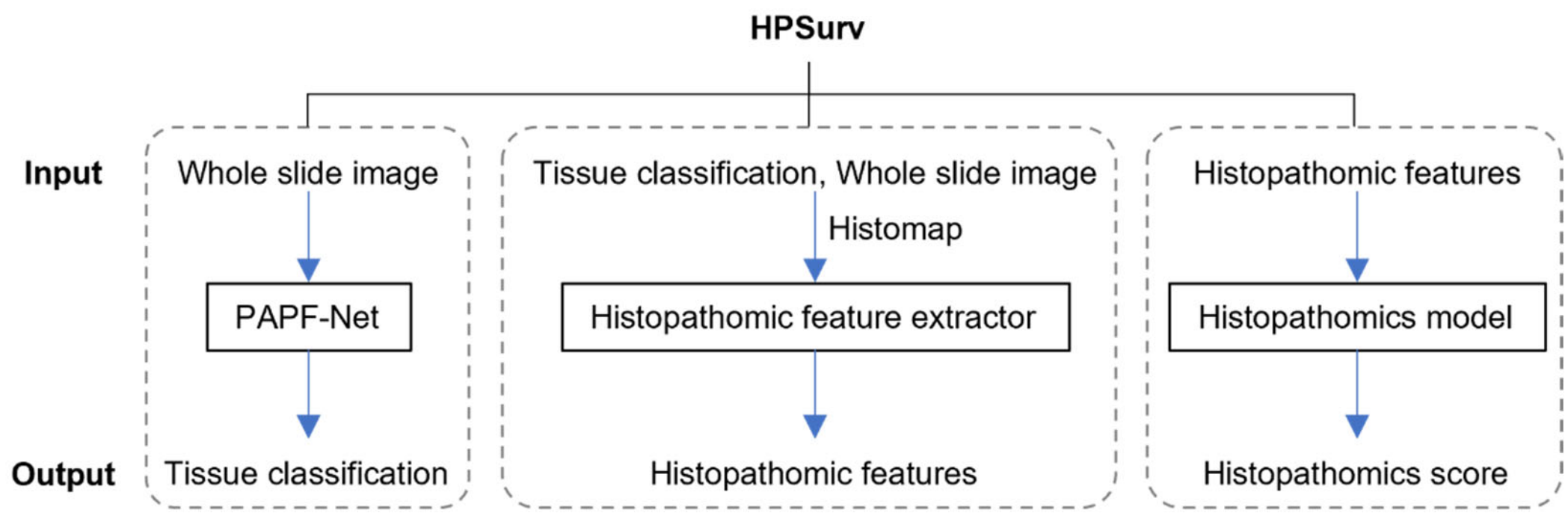

Supplementary Fig.12. Introduction to HPSurv and its key components.

Supplementary Tables

Supplementary Table 1. Patient characteristics

		Overall	PUMCH		FHHMU	PLAGH	NHGH	TCGA-PDAC	P-Value
			PC	VC	ExTC1	ExTC2	ExTC3	ExTC4	
**Number of patients, n**		1020	494	124	136	87	48	131	
**Age, mean (SD)**		61.0 (9.5)	61.0 (9.5)	60.4 (9.4)	60.0 (7.5)	58.9 (9.3)	58.9 (9.7)	64.6 (11.0)	<0.001
**Event, n (%)**	**6-month**	92 (9.0)	33 (6.7)	6 (4.8)	21 (15.4)	5 (5.7)	9 (18.8)	18 (13.7)	<0.001
	**12-month**	281 (28.4)	105 (21.9)	30 (24.4%)	73 (53.7)	13 (15.7)	20 (41.7)	40 (33.6)	<0.001
	**24-month**	479 (52.0)	200 (44.3)	64 (55.7)	102 (75.0)	35 (44.7)	26 (54.1)	70 (72.9)	<0.001
	**36-month**	570 (68.8)	244 (62.1)	73 (70.9)	105 (77.2)	48 (64.0)	27 (71.1)	73 (88.0)	<0.001
**Gender, n (%)**	**Female**	445 (43.6)	213 (43.1)	59 (47.6)	63 (46.3)	33 (37.9)	22 (45.8)	55 (42.0)	0.757
	**Male**	575 (56.4)	281 (56.9)	65 (52.4)	73 (53.7)	54 (62.1)	26 (54.2)	76 (58.0)	
**Lymphonode invasion, n (%)**	**Non-invasion**	475 (46.9)	202 (40.9)	60 (48.8)	97 (71.3)	52 (59.8)	32 (72.7)	32 (24.8)	<0.001
	**Invasion**	538 (53.1)	292 (59.1)	63 (51.2)	39 (28.7)	35 (40.2)	12 (27.3)	97 (75.2)	
**Adjuvant chemotherapy, n (%)**	**Not reciving**	290 (32.8)	83 (21.2)	13 (12.9)	123 (90.4)	18 (22.5)	31 (64.6)	22 (17.5)	<0.001
	**Reciving**	593 (67.2)	309 (78.8)	88 (87.1)	13 (9.6)	62 (77.5)	17 (35.4)	104 (82.5)	
**Neuron invasion, n (%)**	**Non-invasion**	212 (24.0)	148 (30.0)	34 (27.6)	21 (15.4)	5 (5.7)	4 (8.9)		<0.001
	**Invasion**	673 (76.0)	346 (70.0)	89 (72.4)	115 (84.6)	82 (94.3)	41 (91.1)		
**Intravascular tumor thrombus, n (%)**	**Without**	613 (69.4)	318 (64.4)	79 (64.2)	129 (94.9)	57 (65.5)	30 (69.8)		<0.001
	**With**	270 (30.6)	176 (35.6)	44 (35.8)	7 (5.1)	30 (34.5)	13 (30.2)		
**Resction boarder, n (%)**	**Positive**	197 (19.5)	114 (23.1)	30 (24.4)	3 (2.2)	4 (4.6)	1 (2.1)	45 (37.2)	<0.001
	**Negative**	812 (80.5)	380 (76.9)	93 (75.6)	133 (97.8)	83 (95.4)	47 (97.9)	76 (62.8)	
**Pathological T stage, n (%)**	**T1**	180 (20.3)	99 (20.1)	29 (23.4)	30 (22.1)	10 (11.5)	12 (25.0)		0.139
	**T2**	502 (56.6)	284 (57.7)	65 (52.4)	76 (55.9)	52 (59.8)	25 (52.1)		
	**T3**	182 (20.5)	93 (18.9)	24 (19.4)	30 (22.1)	24 (27.6)	11 (22.9)		
	**T4**	23 (2.6)	16 (3.3)	6 (4.8)		1 (1.1)			

**Pathological N stage, n (%)**	**N0**	479 (47.1)	202 (40.9)	60 (48.8)	97 (71.3)	52 (59.8)	36 (75.0)	32 (24.8)	<0.001
	**N1**	379 (37.3)	213 (43.1)	44 (35.8)	29 (21.3)	31 (35.6)	11 (22.9)	51 (39.5)	
	**N2**	159 (15.6)	79 (16.0)	19 (15.4)	10 (7.4)	4 (4.6)	1 (2.1)	46 (35.7)	
**Pathological M stage, n (%)**	**M0**	960 (94.1)	494 (100.0)	124 (100.0)	136 (100.0)	87 (100.0)	48 (100.0)	71 (54.2)	1
	**MX**	60 (5.9)						60 (45.8)	
**AJCC-8th edition stage, n (%)**	**1A**	100 (11.3)	50 (10.1)	14 (11.4)	26 (19.1)	4 (4.6)	6 (12.5)		<0.001
	**1B**	241 (27.2)	104 (21.1)	30 (24.4)	53 (39.0)	33 (37.9)	21 (43.8)		
	**2A**	100 (11.3)	44 (8.9)	15 (12.2)	18 (13.2)	14 (16.1)	9 (18.8)		
	**2B**	318 (35.9)	204 (41.4)	42 (34.1)	29 (21.3)	31 (35.6)	12 (25.0)		
	**3**	128 (14.4)	91 (18.5)	22 (17.9)	10 (7.4)	5 (5.7)			

Note: Significance of average was tested by a one-way anova. The Chi-square test was used to test the significant differences in clinical parameters other than age between different centers. When the theoretical frequency in the contingency table is less than 1 or 5, Fisher's exact test was uesed instead of the Chi-square test.

Supplementary Table 2. Multicenter follow-up information statistics

	PC	VC	ExTC1	ExTC2	ExTC3	ExTC4
**Median survival time (month)**	31	23	11	29	19	20
**Median follow-up time (month)**	47	46	74	65	38	25

Supplementary Table 3. Histological categories of pancreatic tumor

Index	Definition	Abbreviation
0	Foreground of whole amount slide	FWAS
1	Cancer or high-grade pancreatic intraepithelial neoplasia	Ca&HPIN
2	Low-grade pancreatic intraepithelial neoplasia	LPIN
3	Normal tissue	NORM
4	Acinar atrophy or catheterization	Aa&C
5	Tumor stroma	TUMST
6	Immune infiltration	IMMI
7	Lymph node	LYN
8	Nerve	NERV
9	Blood vessel	BV
10	Blood cell aggregation	BCA
11	Necrosis	NEC
12	Other tissues	OTH

Supplementary Table 4. Performance of PAPF-Net for pancreatic tumor tissue classification

ID	Category	AUC	ACC	SEN	SPE
0	Ca&HPIN	0.973 [0.971-0.974]	0.861 [0.844-0.877]	0.965 [0.946-0.983]	0.851 [0.831-0.872]
1	LPIN	1.0 [1.0-1.0]	0.99 [0.988-0.992]	0.997 [0.995-0.999]	0.989 [0.987-0.991]
2	NORM	1.0 [1.0-1.0]	0.997 [0.996-0.998]	0.997 [0.996-0.999]	0.997 [0.996-0.998]
3	Aa&C	1.0 [1.0-1.0]	0.999 [0.999-1.0]	0.995 [0.993-0.997]	1.0 [0.999-1.0]
4	TUMST	1.0 [1.0-1.0]	0.997 [0.997-0.998]	1.0 [0.999-1.0]	0.997 [0.996-0.998]
5	IMMI	0.999 [0.999-0.999]	0.976 [0.97-0.982]	0.992 [0.986-0.998]	0.975 [0.968-0.981]
6	LYN	0.988 [0.987-0.989]	0.939 [0.934-0.945]	0.947 [0.938-0.955]	0.938 [0.932-0.945]
7	NERV	0.988 [0.985-0.99]	0.989 [0.986-0.992]	0.956 [0.95-0.963]	0.992 [0.988-0.995]
8	BV	0.997 [0.997-0.998]	0.974 [0.966-0.982]	0.975 [0.966-0.984]	0.974 [0.964-0.984]
9	BCA	0.999 [0.999-1.0]	0.993 [0.992-0.994]	0.998 [0.996-0.999]	0.993 [0.992-0.994]
10	NEC	1.0 [1.0-1.0]	0.998 [0.998-0.999]	1.0 [0.999-1.0]	0.998 [0.997-0.999]
11	OTH	1.0 [1.0-1.0]	0.997 [0.996-0.998]	0.999 [0.999-1.0]	0.997 [0.996-0.998]

Note：Ca&HPIN, cancer or high-grade pancreatic intraepithelial neoplasia; LPIN, low-grade pancreatic intraepithelial neoplasia; NORM, normal tissue; Aa&C, acinar atrophy or catheterization; TUMST, tumor stroma; IMMI, immune infiltration; LYN, lymph node; NERV, nerve; BV, blood vessel; BCA, blood cell aggregation; NEC, necrosis; OTH, other tissues; AUC, area under the curve; ACC, accuracy; SEN, sensitivity; SPE, specificity.

Supplementary Table 5. Comparison of backbone for tissue classification

**Backbone**	**ACC**
VGG-16	0.873
VGG-19	0.855
ResNet101	0.794
Densenet-169	0.883
ViT	0.841
PLIP transferlearning	0.911
UNI transferlearning	0.938
H-optimus-0	0.915
Phikon-v2	0.922

Supplementary Table 6. Performance of histopathomics model for PDAC overall survival prediction

		PC	VC	ExTC1	EXTC2	ExTC3	ExTC4
**C-index**		0.750 [0.718-0.783]	0.726 [0.658-0.795]	0.823 [0.769-0.878]	0.712 [0.632-0.792]	0.764 [0.656-0.873]	0.782 [0.719-0.846]
**Within 6 months**	**AUC**	0.994 [0.989-1.000]	0.999 [0.993-1.000]	0.920 [0.835-1.000]	0.936 [0.874-0.998]	0.902 [0.815-0.990]	0.922 [0.847-0.997]
**Within 6 months**	**ACC**	0.988 [0.979-0.998]	0.995 [0.979-1.010]	0.884 [0.806-0.962]	0.891 [0.794-0.988]	0.856 [0.752-0.961]	0.903 [0.768-1.000]
**Within 12 months**	**AUC**	0.975 [0.964-0.986]	0.897 [0.842-0.952]	0.896 [0.839-0.953]	0.899 [0.828-0.971]	0.822 [0.691-0.953]	0.870 [0.796-0.944]
**Within 12 months**	**ACC**	0.910 [0.881-0.938]	0.853 [0.785-0.920]	0.875 [0.818-0.932]	0.821 [0.700-0.942]	0.853 [0.756-0.950]	0.855 [0.788-0.923]
**Within 18 months**	**AUC**	0.887 [0.858-0.916]	0.817 [0.742-0.891]	0.883 [0.812-0.953]	0.839 [0.742-0.937]	0.819 [0.690-0.948]	0.836 [0.753-0.918]
**Within 18 months**	**ACC**	0.763 [0.695-0.831]	0.775 [0.702-0.848]	0.889 [0.835-0.944]	0.857 [0.775-0.939]	0.835 [0.732-0.938]	0.818 [0.745-0.892]
**Within 24 months**	**AUC**	0.788 [0.745-0.831]	0.676 [0.570-0.782]	0.823 [0.728-0.919]	0.810 [0.707-0.912]	0.776 [0.633-0.920]	0.776 [0.656-0.896]
**Within 24 months**	**ACC**	0.721 [0.661-0.780]	0.720 [0.641-0.800]	0.837 [0.762-0.912]	0.825 [0.739-0.910]	0.783 [0.667-0.899]	0.783 [0.659-0.908]
**Within 36 months**	**AUC**	0.700 [0.648-0.753]	0.633 [0.509-0.758]	0.795 [0.695-0.896]	0.664 [0.525-0.802]	0.717 [0.502-0.932]	0.815 [0.636-0.994]
**Within 36 months**	**ACC**	0.625 [0.544-0.707]	0.715 [0.507-0.923]	0.816 [0.731-0.900]	0.726 [0.615-0.837]	0.769 [0.615-0.922]	0.870 [0.673-1.067]

Supplementary Table 7. Time-dependent analysis of prediction from histopathomics model for overall survival

Time interval	Metric	PC	VC	ExTC1	EXTC2	ExTC3	ExTC4
**Within 6 months**	**SEN**	1.000 [1.000-1.000]	1.000 [1.000-1.000]	0.921 [0.801-1.042]	0.999 [0.977-1.021]	0.996 [0.952-1.040]	0.855 [0.670-1.039]
	**SPE**	0.988 [0.978-0.998]	0.994 [0.978-1.011]	0.878 [0.783-0.972]	0.884 [0.780-0.988]	0.825 [0.696-0.953]	0.911 [0.740-1.082]
	**PPV**	0.852 [0.739-0.964]	0.918 [0.681-1.156]	0.588 [0.372-0.804]	0.372 [0.084-0.660]	0.566 [0.312-0.820]	0.675 [0.289-1.061]
	**NPV**	1.000 [1.000-1.000]	1.000 [1.000-1.000]	0.984 [0.959-1.009]	1.000 [0.998-1.002]	0.999 [0.986-1.011]	0.976 [0.943-1.008]
**Within 12 months**	**SEN**	0.996 [0.975-1.018]	0.989 [0.948-1.031]	0.891 [0.815-0.966]	0.966 [0.855-1.077]	0.850 [0.692-1.007]	0.848 [0.731-0.964]
	**SPE**	0.885 [0.846-0.924]	0.809 [0.718-0.900]	0.857 [0.771-0.943]	0.794 [0.642-0.946]	0.855 [0.715-0.996]	0.859 [0.760-0.958]
	**PPV**	0.710 [0.628-0.791]	0.625 [0.478-0.772]	0.878 [0.800-0.956]	0.478 [0.249-0.707]	0.808 [0.631-0.985]	0.761 [0.620-0.901]
	**NPV**	0.999 [0.993-1.005]	0.996 [0.979-1.012]	0.871 [0.785-0.957]	0.992 [0.967-1.017]	0.889 [0.771-1.008]	0.916 [0.849-0.982]
**Within 18 months**	**SEN**	0.925 [0.744-1.107]	0.952 [0.859-1.045]	0.936 [0.864-1.009]	0.967 [0.904-1.029]	0.796 [0.618-0.974]	0.921 [0.790-1.052]
	**SPE**	0.678 [0.489-0.867]	0.661 [0.526-0.796]	0.798 [0.673-0.922]	0.782 [0.657-0.908]	0.871 [0.722-1.019]	0.708 [0.546-0.871]
	**PPV**	0.611 [0.474-0.749]	0.645 [0.531-0.759]	0.901 [0.836-0.965]	0.752 [0.614-0.890]	0.849 [0.684-1.015]	0.775 [0.660-0.890]
	**NPV**	0.955 [0.860-1.049]	0.958 [0.885-1.032]	0.868 [0.732-1.004]	0.972 [0.918-1.026]	0.827 [0.678-0.976]	0.900 [0.762-1.037]
**Within 24 months**	**SEN**	0.651 [0.304-0.999]	0.951 [0.750-1.152]	0.870 [0.764-0.977]	0.910 [0.807-1.014]	0.743 [0.532-0.955]	0.808 [0.608-1.008]
	**SPE**	0.776 [0.412-1.139]	0.434 [0.195-0.672]	0.736 [0.564-0.908]	0.750 [0.603-0.898]	0.831 [0.618-1.044]	0.712 [0.477-0.948]
	**PPV**	0.739 [0.547-0.931]	0.680 [0.575-0.784]	0.909 [0.848-0.970]	0.761 [0.626-0.895]	0.845 [0.676-1.014]	0.893 [0.809-0.976]
	**NPV**	0.768 [0.554-0.981]	0.932 [0.680-1.183]	0.665 [0.460-0.871]	0.907 [0.804-1.010]	0.736 [0.545-0.928]	0.585 [0.341-0.829]
**Within 36 months**	**SEN**	0.473 [0.193-0.753]	0.807 [0.360-1.254]	0.848 [0.720-0.975]	0.805 [0.508-1.101]	0.776 [0.496-1.056]	0.887 [0.653-1.120]
	**SPE**	0.883 [0.598-1.168]	0.488 [0.038-0.939]	0.708 [0.515-0.901]	0.590 [0.231-0.949]	0.754 [0.393-1.115]	0.752 [0.466-1.038]
	**PPV**	0.893 [0.779-1.008]	0.811 [0.686-0.936]	0.908 [0.847-0.969]	0.786 [0.650-0.921]	0.892 [0.750-1.034]	0.964 [0.919-1.010]
	**NPV**	0.523 [0.281-0.765]	0.675 [0.178-1.171]	0.600 [0.363-0.837]	0.703 [0.330-1.077]	0.636 [0.232-1.041]	0.552 [0.162-0.942]

Note: SEN, sensitivity; SPE, specificity; PPV, positive predictive value; PPV, negative predictive value.

Supplementary Table 8. Impact of color normalization on performance.

	**With color normalization**		**Without color normalization**		**Batch effects**				
	C-index	6-month AUC	C-index	6-month AUC	C-index (1)	C-index (2)	C-index (3)	C-index (4)	C-index (5)
**VC**	0.726 [0.658-0.795]	0.999 [0.993-1.000]	0.715 [0.644-0.786]	0.999 [0.994-1.000]	0.727 [0.657-0.796]	0.727 [0.660-0.793]	0.723 [0.652-0.795]	0.725 [0.656-0.794]	0.728 [0.657-0.799]
**ExTC1**	0.823 [0.769-0.878]	0.920 [0.835-1.000]	0.729 [0.664-0.794]	0.781 [0.651-0.911]	0.825 [0.770-0.879]	0.825 [0.772-0.878]	0.823 [0.768-0.877]	0.824 [0.769-0.880]	0.824 [0.768-0.879]
**EXTC2**	0.712 [0.632-0.792]	0.936 [0.874-0.998]	0.682 [0.594-0.771]	0.953 [0.904-1.000]	0.709 [0.630-0.787]	0.711 [0.631-0.790]	0.710 [0.626-0.795]	0.709 [0.626-0.792]	0.710 [0.630-0.789]
**ExTC3**	0.764 [0.656-0.873]	0.902 [0.815-0.990]	0.702 [0.629-0.774]	0.754 [0.614-0.894]	0.781 [0.717-0.844]	0.779 [0.715-0.844]	0.782 [0.719-0.845]	0.780 [0.719-0.841]	0.779 [0.716-0.842]
**ExTC4**	0.782 [0.719-0.846]	0.922 [0.847-0.997]	0.701 [0.578-0.824]	0.823 [0.658-0.988]	0.761 [0.654-0.867]	0.764 [0.657-0.871]	0.763 [0.651-0.875]	0.764 [0.657-0.871]	0.761 [0.652-0.871]

Supplementary Table 9. Benchmarking against baseline approaches for overall survival prediction

	**VC**	**ExTC1**	**EXTC2**	**ExTC3**	**ExTC4**
Cox proportional hazards model	0.707 [0.632-0.782]	0.699 [0.640-0.758]	0.622 [0.527-0.716]	0.676 [0.601-0.752]	0.580 [0.458-0.703]
XBGOOST	0.705 [0.633-0.776]	0.722 [0.663-0.781]	0.696 [0.614-0.778]	0.663 [0.585-0.741]	0.682 [0.557-0.806]

Supplementary Table 10a. Univariate analysis of clinical factors for PDAC overall survival

	PC			VC			ExTC1		
	HR (95% CI for HR)	p	C-index	HR (95% CI for HR)	p	C-index	HR (95% CI for HR)	p	C-index
**Gender**	1.1 (0.86-1.4)	0.5	0.5	1.6 (1-2.6)	0.031	0.55	1.1 (0.79-1.6)	0.48	0.53
**Age**	1 (1-1)	0.027	0.55	1 (0.98-1)	0.86	0.52	1 (0.98-1)	0.68	0.51
**Lymphonode invasion**	1.9 (1.4-2.4)	<0.001	0.57	1.2 (0.78-1.9)	0.38	0.52	1.8 (1.2-2.7)	0.003	0.56
**Neuron invasion**	1.3 (1-1.7)	0.035	0.53	1.2 (0.72-2)	0.51	0.53	2.3 (1.3-4.2)	0.007	0.54
**Intravascular tumor thrombus**	1.4 (1.1-1.8)	0.003	0.55	1.2 (0.75-1.9)	0.44	0.53	0.96 (0.42-2.2)	0.93	0.5
**R0 resection**	0.75 (0.57-0.98)	0.033	0.53	0.62 (0.38-1)	0.055	0.56	0.11 (0.032-0.35)	<0.001	0.52
**Pathological T stage**	1.3 (1.1-1.5)	0.002	0.55	1.2 (0.88-1.5)	0.3	0.54	1.1 (0.86-1.5)	0.37	0.54
**Pathological N stage**	1.6 (1.3-1.8)	<0.001	0.58	1.1 (0.85-1.5)	0.37	0.52	1.4 (1.1-1.8)	0.019	0.55
**Adjuvant single-drug or doublet chemotherapy**	0.46 (0.35-0.62)	<0.001	0.58	0.56 (0.28-1.1)	0.098	0.53	0.78 (0.41-1.5)	0.45	0.51
**AJCC-8th edition stage**	1.3 (1.2-1.4)	<0.001	0.59	1.1 (0.94-1.3)	0.23	0.53	1.2 (1-1.4)	0.026	0.55

Supplementary Table 10b. Univariate analysis of clinical factors for PDAC overall survival

	ExTC2			ExTC3			ExTC4		
	HR (95% CI for HR)	p	C-index	HR (95% CI for HR)	p	C-index	HR (95% CI for HR)	p	C-index
**Gender**	1.1 (0.63-1.8)	0.81	0.52	0.51 (0.24-1.1)	0.087	0.57	0.51 (0.24-1.1)	0.087	0.57
**Age**	1 (0.98-1)	0.57	0.55	1 (0.99-1.1)	0.13	0.57	1 (0.99-1.1)	0.13	0.57
**Lymphonode invasion**	0.92 (0.54-1.6)	0.75	0.53	3.2 (1.4-7.3)	0.005	0.61	3.2 (1.4-7.3)	0.005	0.61
**Neuron invasion**	7.7 (1.1-56)	0.044	0.55	0.93 (0.22-4)	0.93	0.51	0.93 (0.22-4)	0.93	0.51
**Intravascular tumor thrombus**	2.5 (1.4-4.2)	<0.001	0.61	2.7 (1.2-6.2)	0.017	0.6	2.7 (1.2-6.2)	0.017	0.6
**R0 resection**	1.4 (0.35-5.9)	0.62	0.52	0.13 (0.016-1)	0.055	0.52	0.13 (0.016-1)	0.055	0.52
**Pathological T stage**	1.1 (0.72-1.7)	0.68	0.52	1.2 (0.67-2)	0.6	0.55	1.2 (0.67-2)	0.6	0.55
**Pathological N stage**	1 (0.64-1.6)	0.94	0.47	1.5 (0.68-3.4)	0.31	0.52	1.5 (0.68-3.4)	0.31	0.52
**Adjuvant single-drug or doublet chemotherapy**	0.48 (0.26-0.88)	0.017	0.6	0.29 (0.11-0.76)	0.012	0.63	0.29 (0.11-0.76)	0.012	0.63
**AJCC-8th edition stage**	0.99 (0.76-1.3)	0.91	0.52	1.2 (0.85-1.8)	0.28	0.55	1.2 (0.85-1.8)	0.28	0.55

Supplementary Table 11. Performance comparison of HPsurv and clinical prognostic indicators for overall survival (OS) prediction in the primary cohort

		HPSurv	AJCC-8th edition stage	Lymphonode invasion	Neuron invasion	Intravascular tumor thrombus
**C-index**		0.750 [0.718-0.783]	0.594 [0.561-0.627]	0.572 [0.541-0.602]	0.525 [0.497-0.554]	0.554 [0.524-0.584]
**Death within 6 months**	**AUC**	0.994 [0.989-1.000]	0.603 [0.521-0.686]	0.588 [0.509-0.667]	0.499 [0.417-0.581]	0.586 [0.495-0.677]
	**ACC**	0.988 [0.979-0.998]	0.401 [0.162-0.641]	0.452 [0.316-0.588]	0.641 [0.057-1.225]	0.656 [0.544-0.769]
**Death within 12 months**	**AUC**	0.975 [0.964-0.986]	0.599 [0.540-0.657]	0.558 [0.506-0.610]	0.506 [0.456-0.557]	0.574 [0.522-0.626]
	**ACC**	0.910 [0.881-0.938]	0.542 [0.287-0.796]	0.499 [0.416-0.582]	0.555 [0.192-0.917]	0.634 [0.591-0.677]
**Death within 18 months**	**AUC**	0.887 [0.858-0.916]	0.610 [0.560-0.661]	0.575 [0.530-0.621]	0.534 [0.490-0.578]	0.592 [0.544-0.639]
	**ACC**	0.763 [0.695-0.831]	0.567 [0.445-0.689]	0.541 [0.496-0.586]	0.486 [0.389-0.583]	0.630 [0.584-0.676]
**Death within 24 months**	**AUC**	0.788 [0.745-0.831]	0.630 [0.581-0.680]	0.612 [0.568-0.656]	0.566 [0.524-0.607]	0.573 [0.529-0.617]
	**ACC**	0.721 [0.661-0.780]	0.601 [0.553-0.650]	0.602 [0.557-0.647]	0.545 [0.499-0.590]	0.589 [0.544-0.633]
**Death within 36 months**	**AUC**	0.700 [0.648-0.753]	0.662 [0.608-0.716]	0.621 [0.571-0.671]	0.563 [0.514-0.611]	0.581 [0.537-0.626]
	**ACC**	0.625 [0.544-0.707]	0.638 [0.568-0.708]	0.636 [0.588-0.684]	0.607 [0.551-0.664]	0.538 [0.490-0.586]

Note: Values are presented as mean [95% confidence interval].

Supplementary Table 12. Performance comparison of HPsurv and clinical prognostic indicators for overall survival (OS) prediction in the validation cohort

		HPSurv	AJCC-8th edition stage	Lymphonode invasion	Neuron invasion	Intravascular tumor thrombus
**C-index**		0.726 [0.658-0.795]	0.538 [0.474-0.603]	0.517 [0.454-0.580]	0.527 [0.472-0.581]	0.523 [0.466-0.580]
**Death within 6 months**	**AUC**	0.999 [0.993-1.000]	0.474 [0.256-0.693]	0.405 [0.187-0.624]	0.379 [0.147-0.611]	0.493 [0.283-0.702]
	**ACC**	0.995 [0.979-1.010]	0.330 [-0.123-0.782]	0.871 [0.528-1.213]	0.859 [0.412-1.307]	0.813 [0.507-1.119]
**Death within 12 months**	**AUC**	0.897 [0.842-0.952]	0.512 [0.398-0.626]	0.493 [0.387-0.600]	0.528 [0.440-0.617]	0.463 [0.369-0.557]
	**ACC**	0.853 [0.785-0.920]	0.546 [0.245-0.847]	0.653 [0.418-0.888]	0.510 [0.210-0.809]	0.720 [0.571-0.869]
**Death within 18 months**	**AUC**	0.817 [0.742-0.891]	0.590 [0.488-0.692]	0.556 [0.465-0.647]	0.574 [0.497-0.652]	0.588 [0.502-0.673]
	**ACC**	0.775 [0.702-0.848]	0.568 [0.464-0.671]	0.564 [0.481-0.647]	0.531 [0.443-0.619]	0.620 [0.539-0.701]
**Death within 24 months**	**AUC**	0.676 [0.570-0.782]	0.553 [0.451-0.656]	0.520 [0.426-0.614]	0.538 [0.457-0.619]	0.560 [0.474-0.646]
	**ACC**	0.720 [0.641-0.800]	0.561 [0.453-0.670]	0.512 [0.392-0.632]	0.554 [0.429-0.678]	0.538 [0.436-0.641]
**Death within 36 months**	**AUC**	0.633 [0.509-0.758]	0.568 [0.447-0.689]	0.543 [0.433-0.653]	0.524 [0.427-0.620]	0.546 [0.451-0.642]
	**ACC**	0.715 [0.507-0.923]	0.585 [0.329-0.841]	0.497 [0.264-0.731]	0.524 [0.192-0.855]	0.448 [0.279-0.617]

Note: Values are presented as mean [95% confidence interval].

Supplementary Table 13. Performance comparison of HPsurv and clinical prognostic indicators for overall survival (OS) prediction in the external testing cohort 1

		HPSurv	AJCC-8th edition stage	Lymphonode invasion	Neuron invasion	Intravascular tumor thrombus
**C-index**		0.823 [0.769-0.878]	0.552 [0.495-0.609]	0.558 [0.514-0.602]	0.537 [0.493-0.581]	0.503 [0.477-0.529]
**Death within 6 months**	**AUC**	0.920 [0.835-1.000]	0.544 [0.405-0.683]	0.557 [0.439-0.675]	0.535 [0.464-0.605]	0.526 [0.458-0.594]
	**ACC**	0.884 [0.806-0.962]	0.641 [0.498-0.784]	0.715 [0.572-0.858]	0.387 [-0.039-0.813]	0.834 [0.772-0.896]
**Death within 12 months**	**AUC**	0.896 [0.839-0.953]	0.557 [0.465-0.650]	0.561 [0.488-0.634]	0.518 [0.458-0.578]	0.489 [0.451-0.527]
	**ACC**	0.875 [0.818-0.932]	0.559 [0.479-0.640]	0.545 [0.462-0.628]	0.529 [0.416-0.643]	0.469 [0.386-0.552]
**Death within 18 months**	**AUC**	0.883 [0.812-0.953]	0.623 [0.535-0.711]	0.601 [0.526-0.676]	0.548 [0.481-0.616]	0.490 [0.448-0.531]
	**ACC**	0.889 [0.835-0.944]	0.561 [0.456-0.666]	0.521 [0.434-0.608]	0.634 [0.453-0.815]	0.346 [0.268-0.424]
**Death within 24 months**	**AUC**	0.823 [0.728-0.919]	0.590 [0.491-0.689]	0.591 [0.516-0.667]	0.592 [0.508-0.676]	0.495 [0.448-0.542]
	**ACC**	0.837 [0.762-0.912]	0.498 [0.335-0.661]	0.461 [0.366-0.557]	0.737 [0.605-0.869]	0.267 [0.185-0.350]
**Death within 36 months**	**AUC**	0.795 [0.695-0.896]	0.605 [0.504-0.707]	0.625 [0.554-0.695]	0.609 [0.521-0.698]	0.492 [0.444-0.540]
	**ACC**	0.816 [0.731-0.900]	0.489 [0.366-0.611]	0.471 [0.385-0.558]	0.761 [0.657-0.865]	0.245 [0.168-0.321]

Note: Values are presented as mean [95% confidence interval].

Supplementary Table 14. Performance comparison of HPsurv and clinical prognostic indicators for overall survival (OS) prediction in the external testing cohort 2

		HPSurv	AJCC-8th edition stage	Lymphonode invasion	Neuron invasion	Intravascular tumor thrombus
**C-index**		0.712 [0.632-0.792]	0.479 [0.403-0.555]	0.466 [0.399-0.534]	0.547 [0.511-0.583]	0.610 [0.544-0.676]
**Death within 6 months**	**AUC**	0.936 [0.874-0.998]	0.448 [0.241-0.655]	0.394 [0.193-0.594]	0.531 [0.504-0.558]	0.632 [0.386-0.879]
	**ACC**	0.891 [0.794-0.988]	0.295 [-0.091-0.680]	0.891 [0.646-1.136]	0.121 [-0.019-0.262]	0.713 [0.499-0.927]
**Death within 12 months**	**AUC**	0.899 [0.828-0.971]	0.512 [0.355-0.670]	0.441 [0.300-0.581]	0.536 [0.506-0.566]	0.560 [0.410-0.709]
	**ACC**	0.821 [0.700-0.942]	0.498 [0.041-0.955]	0.788 [0.573-1.004]	0.223 [0.096-0.350]	0.693 [0.508-0.878]
**Death within 18 months**	**AUC**	0.839 [0.742-0.937]	0.396 [0.272-0.519]	0.408 [0.298-0.519]	0.553 [0.509-0.598]	0.652 [0.539-0.765]
	**ACC**	0.857 [0.775-0.939]	0.569 [0.404-0.735]	0.596 [0.487-0.704]	0.468 [0.360-0.576]	0.673 [0.566-0.780]
**Death within 24 months**	**AUC**	0.810 [0.707-0.912]	0.395 [0.272-0.518]	0.408 [0.299-0.517]	0.563 [0.508-0.617]	0.685 [0.583-0.787]
	**ACC**	0.825 [0.739-0.910]	0.550 [0.429-0.671]	0.533 [0.421-0.644]	0.533 [0.418-0.648]	0.692 [0.591-0.794]
**Death within 36 months**	**AUC**	0.664 [0.525-0.802]	0.480 [0.357-0.603]	0.479 [0.359-0.598]	0.594 [0.519-0.668]	0.676 [0.577-0.775]
	**ACC**	0.726 [0.615-0.837]	0.455 [0.255-0.655]	0.412 [0.236-0.588]	0.706 [0.591-0.822]	0.626 [0.516-0.736]

Note: Values are presented as mean [95% confidence interval].

Supplementary Table 15. Performance comparison of HPsurv and clinical prognostic indicators for overall survival (OS) prediction in the external testing cohort 3

		HPSurv	AJCC-8th edition stage	Lymphonode invasion	Neuron invasion	Intravascular tumor thrombus
**C-index**		0.764 [0.656-0.873]	0.550 [0.445-0.655]	0.612 [0.526-0.699]	0.488 [0.416-0.559]	0.609 [0.523-0.695]
**Death within 6 months**	**AUC**	0.902 [0.815-0.990]	0.578 [0.406-0.750]	0.617 [0.426-0.807]	0.417 [0.274-0.560]	0.598 [0.406-0.790]
	**ACC**	0.856 [0.752-0.961]	0.471 [0.142-0.801]	0.748 [0.616-0.880]	0.727 [0.310-1.144]	0.732 [0.590-0.873]
**Death within 12 months**	**AUC**	0.822 [0.691-0.953]	0.521 [0.362-0.681]	0.633 [0.509-0.757]	0.487 [0.406-0.569]	0.649 [0.523-0.774]
	**ACC**	0.853 [0.756-0.950]	0.558 [0.403-0.713]	0.672 [0.544-0.801]	0.531 [0.352-0.711]	0.682 [0.556-0.809]
**Death within 18 months**	**AUC**	0.819 [0.690-0.948]	0.564 [0.411-0.717]	0.634 [0.516-0.751]	0.496 [0.417-0.576]	0.657 [0.533-0.780]
	**ACC**	0.835 [0.732-0.938]	0.581 [0.451-0.710]	0.644 [0.510-0.778]	0.518 [0.383-0.652]	0.666 [0.533-0.799]
**Death within 24 months**	**AUC**	0.776 [0.633-0.920]	0.574 [0.421-0.726]	0.691 [0.583-0.798]	0.507 [0.427-0.587]	0.663 [0.553-0.774]
	**ACC**	0.783 [0.667-0.899]	0.589 [0.451-0.727]	0.670 [0.536-0.804]	0.522 [0.356-0.688]	0.644 [0.511-0.776]
**Death within 36 months**	**AUC**	0.717 [0.502-0.932]	0.590 [0.404-0.775]	0.704 [0.612-0.795]	0.556 [0.427-0.686]	0.703 [0.610-0.796]
	**ACC**	0.769 [0.615-0.922]	0.565 [0.326-0.804]	0.578 [0.422-0.734]	0.631 [0.248-1.014]	0.581 [0.428-0.733]

Note: Values are presented as mean [95% confidence interval].

Supplementary Table 16. Performance comparison of HPsurv and clinical prognostic indicators for overall survival (OS) prediction in the external testing cohort 4

		HPSurv	AJCC-8th edition stage	Lymphonode invasion	Neuron invasion	Intravascular tumor thrombus
**C-index**		0.782 [0.719-0.846]	-	0.556 [0.498-0.613]	0.500 [0.500-0.500]	-
**Death within 6 months**	**AUC**	0.922 [0.847-0.997]	-	0.546 [0.450-0.642]	0.500 [0.500-0.500]	-
	**ACC**	0.903 [0.768-1.000]	-	0.433 [0.046-0.820]	0.861 [0.801-0.921]	-
**Death within 12 months**	**AUC**	0.870 [0.796-0.944]	-	0.548 [0.470-0.626]	0.500 [0.500-0.500]	-
	**ACC**	0.855 [0.788-0.923]	-	0.493 [0.354-0.631]	0.660 [0.574-0.746]	-
**Death within 18 months**	**AUC**	0.836 [0.753-0.918]	-	0.585 [0.505-0.666]	0.500 [0.500-0.500]	-
	**ACC**	0.818 [0.745-0.892]	-	0.592 [0.498-0.686]	0.484 [0.389-0.579]	-
**Death within 24 months**	**AUC**	0.776 [0.656-0.896]	-	0.608 [0.499-0.716]	0.500 [0.500-0.500]	-
	**ACC**	0.783 [0.659-0.908]	-	0.712 [0.540-0.885]	0.257 [0.168-0.346]	-
**Death within 36 months**	**AUC**	0.815 [0.636-0.994]	-	0.621 [0.455-0.787]	0.500 [0.500-0.500]	-
	**ACC**	0.870 [0.673-1.067]	-	0.747 [0.388-1.106]	0.119 [0.049-0.188]	-

Note: Values are presented as mean [95% confidence interval]. "–" indicates not calculable due to missing clinical data.

Supplementary Table 17. Performance of the OS prediction model combining clinical factors with histopathomics score

	PC		VC		ExTC1		ExTC2		ExTC3		ExTC4	
	Concordance with OS	Significant difference	Concordance with OS	Significant difference	Concordance with OS	Significant difference	Concordance with OS	Significant difference	Concordance with OS	Significant difference	Concordance with OS	Significant difference
Clinical model	0.664 [0.647-0.681]	p<0.001	0.585 [0.551-0.619]	p<0.001	0.571 [0.539-0.603]	p<0.001	0.63 [0.587-0.673]	p<0.001	0.684 [0.633-0.735]	p<0.001	0.672 [0.636-0.708]	p<0.001
Clinical factors+histopathomics score	0.727 [0.713-0.741]		0.669 [0.640-0.698]		0.685 [0.657-0.713]		0.689 [0.65-0.728]		0.745 [0.696-0.794]		0.748 [0.718-0.778]	

Note: Values are presented as mean [95% confidence interval]

Supplementary Table 18. Workflow and experimental details for the development and validation of HPsurv

Step	Data	Hyperparameter
Data partitioning	Primary cohort data	None
Development of a pathological tissue classification network (PAPF-Net)	Primary cohort data	Backbone: UNI-based network;<br>Initial learning rate: 0.001;<br>Optimizer: Adam.
Feature selection	PC data	Significance threshold for the Mann－Whitney U test: $P < 0.05$;<br>Time-dependent event time points: [3, 6, 12, 18, 24, 36];<br>Pearson correlation coefficient threshold: $r > 0.95$.
Development of an overall survival (OS) prediction model (HTSurv-Net)	PC data	Initial learning rate: 0.0001;<br>Optimizer: Adam;<br>In the multi-task supervised learning framework, time-dependent event time points were set at [6, 12, 24, 36], and the loss weights for time-dependent event classification and OS risk regression were both set to 0.5.<br>Early stopping criterion: training was terminated if the C-index did not improve for five consecutive epochs.
Validation and testing	All model parameters were fixed after training, and the model was evaluated exclusively on the validation cohort (VC) and external testing cohorts (ExTC1－4).	The cut-off value was determined in the primary cohort (PC) using the Youden index derived from 24-month mortality prediction, to ensure optimal model performance.

Supplementary Table 19. Histopathomic feature description

	Identifier	Feature group	Description
1	{}%all_num	Statistical features	Absolute area of pathological tissue
2	PredValueMean_{}		Mean of predicted value of a single pathological tissue
3	PredValueStd_{}		Variance of predicted values for a single pathological tissue
4	{}%{}_num		The proportion of a single pathological category compared to other tissues
5	x{}y_score		The ratio between the total score of a single pathological tissue category and the total score of other tissues
6	{}%all_num		The proportion of a single category to the total area of whole slide image
7	{}%all_score		The proportion of the predicted score of a single pathological tissue category to the total area of the whole slide image
8	shannon_entropy		Abundance of pathological tissues (Shannon entropy)
9	moments_{}_area	Morphology	Geometric features of the outline of pathological tissue (area)
10	Centroid_{}_x		Geometric features of the outline of pathological tissue (coordinate)
11	perimeter_{}		Perimeter of the pathological tissue
12	Ellipse_w_{}		The major axis of the pathological tissue is approximately elliptical.
13	Ellipse_h_{}		The pathological tissue approximates the minor axis of an ellipse.
14	Ellipse_ang_{}		The rotation angle of the approximately elliptical pathological tissue
15	Circle_w_{}		The radius of the smallest circumcircle of a single pathological tissue
16	convexity_{}		The degree of indentation of the contour of a single pathological tissue
17	ShapeNum_{}		Number of individual pathological tissues
18	glcm_contrast_{}	Texture	Measuring the degree of local variation between pixel grayscale values in pathological tissue images
19	glcm_homogeneity_{}		Measuring the uniformity or similarity of pixel grayscale values in an image
20	glcm_energy_{}		Texture complexity representing pathological tissue images
21	glcm_correlation_{}		To evaluate images, the correlation between pixel pairs in pathological tissue regions is calculated
22	glcm_ASM_{}		Measuring the homogeneity or smoothness of images of pathological tissue regions
23	lbp_{}		A binary pattern is generated by comparing the grayscale values of each pixel with those of its neighboring pixels
